# Degradable Bottlebrush Polypeptides and the Impact of their Architecture on Cell Uptake, Pharmacokinetics, and Biodistribution In Vivo

**DOI:** 10.1002/smll.202300767

**Published:** 2023-02-26

**Authors:** Paul Strasser, Bianca Montsch, Silvia Weiss, Haider Sami, Christoph Kugler, Sonja Hager, Hemma Schueffl, Robert Mader, Oliver Brüggemann, Christian R. Kowol, Manfred Ogris, Petra Heffeter, Ian Teasdale

**Affiliations:** ^1^ Institute of Polymer Chemistry Johannes Kepler University Linz Linz 4040 Austria; ^2^ Center for Cancer Research and Comprehensive Cancer Center Medical University Vienna Vienna 1090 Austria; ^3^ Research Cluster “Translational Cancer Therapy Research” University of Vienna Vienna 1090 Austria; ^4^ Laboratory of Macromolecular Cancer Therapeutics (MMCT) Department of Pharmaceutical Sciences Faculty of Life Sciences University of Vienna Vienna 1090 Austria; ^5^ Department of Food Chemistry and Toxicology Faculty of Chemistry University of Vienna Vienna 1090 Austria; ^6^ Department of Medicine I Medical University of Vienna Vienna 1090 Austria; ^7^ Institute of Inorganic Chemistry Faculty of Chemistry University of Vienna Vienna 1090 Austria

**Keywords:** biodistribution, bottlebrush polymers, nanomedicine, pharmacokinetics, polyglutamate, polymer therapeutics, polyphosphazenes

## Abstract

Bottlebrush polymers are highly promising as unimolecular nanomedicines due to their unique control over the critical parameters of size, shape and chemical function. However, since they are prepared from biopersistent carbon backbones, most known bottlebrush polymers are non‐degradable and thus unsuitable for systemic therapeutic administration. Herein, we report the design and synthesis of novel poly(organo)phosphazene‐*g*‐poly(α‐glutamate) (PPz‐g‐PGA) bottlebrush polymers with exceptional control over their structure and molecular dimensions (Dh ≈ 15–50 nm). These single macromolecules show outstanding aqueous solubility, ultra‐high multivalency and biodegradability, making them ideal as nanomedicines. While well‐established in polymer therapeutics, it has hitherto not been possible to prepare defined single macromolecules of PGA in these nanosized dimensions. A direct correlation was observed between the macromolecular dimensions of the bottlebrush polymers and their intracellular uptake in CT26 colon cancer cells. Furthermore, the bottlebrush macromolecular structure visibly enhanced the pharmacokinetics by reducing renal clearance and extending plasma half‐lives. Real‐time analysis of the biodistribution dynamics showed architecture‐driven organ distribution and enhanced tumor accumulation. This work, therefore, introduces a robust, controlled synthesis route to bottlebrush polypeptides, overcoming limitations of current polymer‐based nanomedicines and, in doing so, offers valuable insights into the influence of architecture on the in vivo performance of nanomedicines.

## Graphical abstract

Nanosized but fully biodegradable bottlebrush polymers are prepared in a well‐defined manner by growing synthetic polypeptides on a polyphosphazene main chain. Such unique materials have substantial potential as tumor‐ and organ‐targeting therapeutics. The size and shape of the macromolecules are demonstrated to define their cell uptake, pharmacokinetics, and ultimate in vivo tissue and organ distribution.

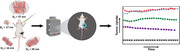

## 1 Introduction

Polymer therapeutics, multi‐component macromolecules covalently linked to an active pharmaceutical ingredient, have proven to be an effective tool for a diverse set of challenges in biomedicine.^[^ ^1^ ^]^ Such macromolecular prodrugs offer a specific set of advantages over small molecules, such as improved solubility, higher stability, prolonged plasma circulation and reduced side effects.^[^ ^2^, ^3^, ^4^ ^]^ Alongside high‐molecular‐weight (MW), macromolecular architecture and conformation in solution are of profound importance in designing effective polymer therapeutics for nanomedicine.^[^ ^2^, ^5^ ^]^ Different 3D macromolecular structures can be realized, ranging from highly symmetric dendrimers^[^ ^6^ ^]^ to hyper‐branched polymers^[^ ^7^ ^]^ and from star‐branched polymers^[^ ^8^ ^]^ to macromolecular bottlebrushes.^[^ ^9^, ^10^ ^]^ The abundance of functional groups, alongside lower viscosity and improved solubility compared to linear polymers, as well as improvements in biodistribution^[^ ^4^ ^]^ makes branched polymer architectures especially suitable for drug‐delivery applications.^[^ ^11^ ^]^ Bottlebrush polymers, in particular, consisting of a linear polymer backbone with densely grafted side chains,^[^ ^12^ ^]^ provide a set of distinct advantages.^[^ ^2^, ^10^, ^13^ ^]^ Due to their unimolecular nature, they are synthetically robust, well‐defined and more stable in solution than, for example, self‐assembled nanoformulations. The high number of side‐chains can positively influence the blood circulation half‐life^[^ ^4^ ^]^ and possibly facilitate penetration of the tumor tissue, resulting in high tumoral drug accumulation.^[^ ^4^, ^12^, ^14^ ^]^

Poly(α‐glutamate) (PGA) is preeminent for polymer therapeutics and has been tested in the form of the drug Opaxio/Polyglumex in several phase III clinical trials (clinical trial identifiers, e.g., NCT00551733 and NCT00269828), in which reduced adverse effects compared to free paclitaxel were reported.^[^ ^15^, ^16^ ^]^ However, due to its low impact on overall survival, Opaxio/Polyglumex has not been clinically approved.^[^ ^15^, ^16^, ^17^ ^]^ Constituted of the endogenous non‐essential amino acid glutamate, PGA is water‐soluble and has wide biocompatibility and biodegradability in a suitable timeframe for therapeutic applications.^[^ ^18^, ^19^, ^20^ ^]^ It stands out in the field as one of very few genuinely degradable, functionalizable water‐soluble synthetic polymers and a promising candidate in the search for alternatives to the ubiquitous but problematic PEGs.^[^ ^21^, ^22^, ^23^, ^24^ ^]^ The polypeptide can be synthesized in a living fashion by the ring‐opening polymerization of α‐*N*‐carboxyanhydrides (NCAs) under various conditions.^[^ ^25^, ^26^, ^27^, ^28^, ^29^ ^]^ Notably, linear synthetic PGAs, such as those used for Polyglumex, are still limited by their achievable MW at reasonable dispersity (Ð). Vicent et al. improved this aspect in the form of star‐branched PGAs, showing increased plasma half‐life time (*t*_1/2_) and cell uptake enhancement.^[^ ^30^ ^]^ This has led to the development of different nanostructures, for example, self‐assembled nanorods^[^ ^31^ ^]^ and sphere‐like cross‐linked nanocarriers.^[^ ^32^ ^]^ These structures, alongside their linear analogues, have been broadly investigated for various applications in biomedicine.^[^ ^33^, ^34^, ^35^, ^36^ ^]^ Nevertheless, the MW of the star‐shaped PGAs is still limited in size, in this case by the small number of arms. Recently, bottlebrush‐type poly(norbornene)‐*graft‐*PGA has been reported.^[^ ^37^ ^]^ However, despite enabling the synthesis of PGA co‐polymers with significantly increased MW and notably low Ð, the norbornene backbone is non‐degradable, restricting its applicability, for example, in systemic drug delivery.

Poly(organo)phosphazenes (PPz) are a versatile class of organic–inorganic hybrid polymers, consisting of a highly flexible inorganic [—P=N—] backbone,^[^ ^38^ ^]^ with manifold applicability in the biomedical field,^[^ ^39^ ^]^ including food and drug administration (FDA)‐approved stent linings,^[^ ^40^ ^]^ drug and protein delivery^[^ ^41^ ^]^ and vaccine adjuvants.^[^ ^42^, ^43^, ^44^ ^]^ Controlled polymerization routes, most prominently via a living cationic polymerization, allow for the synthesis of polymers with controlled length and narrow Ð.^[^ ^45^, ^46^ ^]^ Subsequently, post‐polymerization modification reactions endow PPz with precise adjustability in terms of functionality, degradability, and architecture.^[^ ^39^, ^47^ ^]^ Herein, we report the synthesis of a series of high MW, fully degradable bottlebrush polymers based on a PPz backbone and PGA side chains. The biological properties of the new derivatives were compared to a linear PGA analogue both in cell culture and in vivo. A detailed analysis of their pharmacokinetics, as well as real‐time biodistribution investigations down to the histological level are performed on CT26 colon cancer‐bearing mice.

## 2 Results

### 2.1 Polymer Synthesis and Characterization

**Figure 1:**
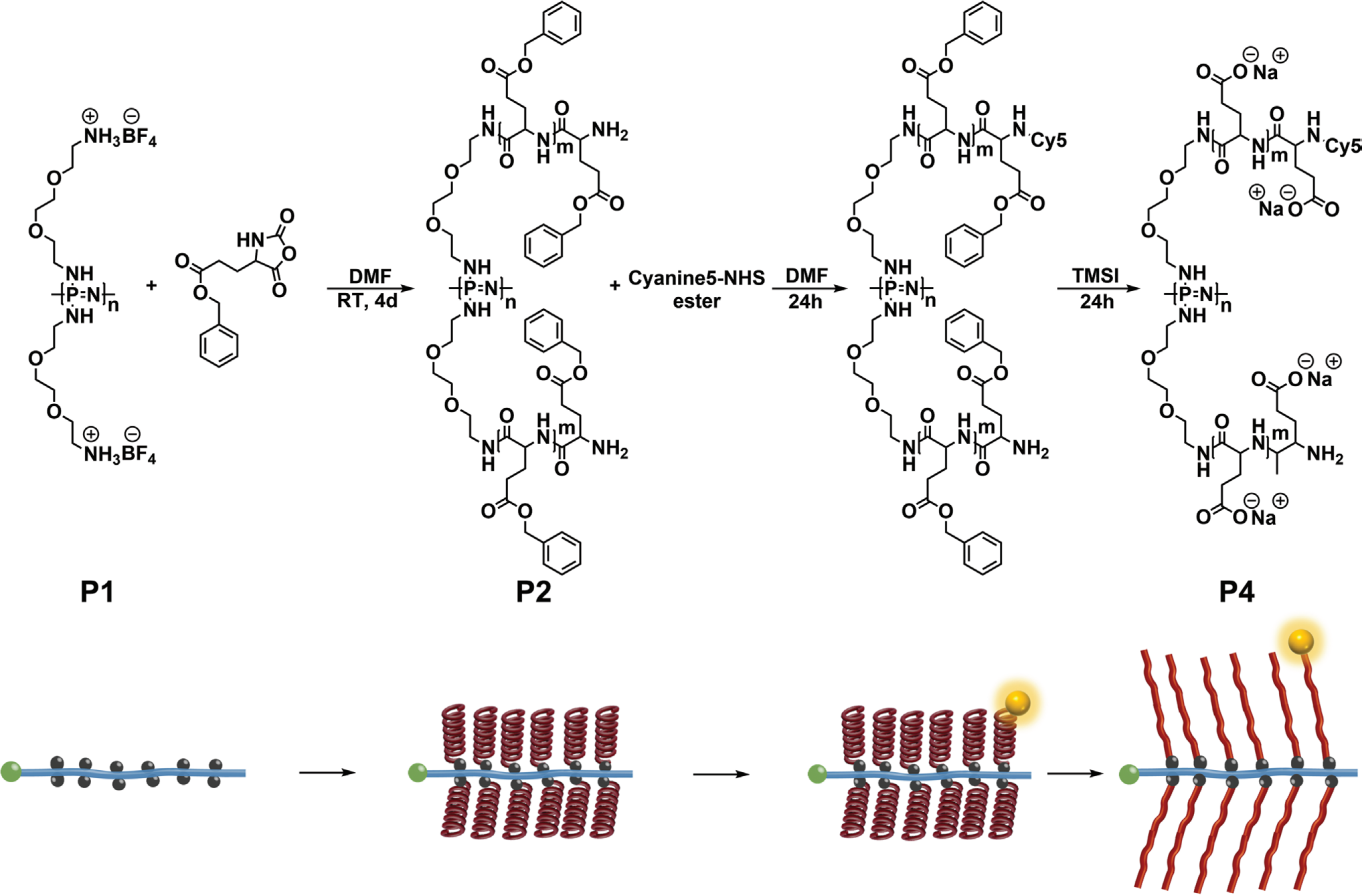
Synthesis of PPz‐*g*‐PGA bottlebrush polymers and their fluorescent labelling with Cy5‐NHS ester at the *N*‐terminus of the PGA grafts. Polymer series P2 correspond to the benzyl‐ester protected macromolecular intermediates, subsequently deprotected and transformed into the final PPz‐*g*‐PGA graft polymers P4.

The controlled polymerization of Cl_3_PNSiMe_3_ initiated by 4‐(diphenylphosphino)styrene was conducted in a ratio M:I of 75:1, giving the macromolecular intermediate poly(dichloro)phosphazene with a chain‐length of (*N*_BB_) 73 repeat units according to ^1^H‐NMR spectroscopy.^[^ ^45^, ^48^ ^]^ Subsequent substitution with mono‐BOC protected 2,2′‐(ethylendioxy)‐bisethylamine resulted in a PPz bearing two pendant amine groups per repeat unit (Figure S1, Supporting Information), which could then be readily converted to a BF_4_ ammonium salt in the macroinitiator P1 (**Figure** **1**) with a conversion of >95%, as confirmed by NMR spectroscopy (Figures S2–S4, Supporting Information).^[^ ^49^ ^]^

Vicent et al. have recently shown BF_4_ ammonium salts to be excellent macroinitiators for the NCA polymerization to PGA.^[^ ^29^, ^30^ ^]^ Indeed, with the PPz macromolecular initiator (P1) in hand, poly(benzyl‐l‐glutamic acid) (PBLGA) side‐chains in various lengths (*N*_SC_) were synthesized (P2.1–2.3) via a grafting‐from approach with *N*‐carboxyanhydride (Figure 1). A linear PBLGA polymer was also prepared (P3) from a dodecylamine initiator (Figures S5 and S6, Supporting Information) to be used as a reference in biological studies (**Table** **1**). After precipitation into cold diethyl ether (Et_2_O), the polymers were characterized without further purification by NMR spectroscopy, circular dichroism (CD) spectroscopy and size‐exclusion chromatography (SEC) in dimethylformamide (DMF), showing an increase in MW with longer PGA side chains (Figure S7, Supporting Information).

**Table 1:** MW (SEC) and DLS analysis of bottlebrush PPz‐*g*‐PGA and linear PGA polymers.

	*N*_BB_ (targeted)	*N*_SC_ (targeted)	GAU	*M_n_*_PBLGA_ [kDa]^a)^	Ð_PBLGA_ ^a)^	*M_n_*_PGA_ [kDa]^b)^	*M_n_*_PGA_ [kDa]^c)^, *	Ð_PGA_ ^c)^, *	Dh _Z‐Average_ [nm]^d)^, *
P4.1	73(75)	2.5(10)	365	69	1.43	91	24	1.39	16
P4.2	73(75)	20(50)	2920	275	1.98	466	231	1.31	35
P4.3	73(75)	87(100)	12 702	991	1.38	1943	248**	1.29	47
P5	–	224(600)	224	22	1.11	34	16	1.09	17

^a)^ Determined by SEC in DMF (10 mm LiBr) and multiple detector calibration with polystyrene standard, 100% mass recovery assumed

^b)^ Determined by ^1^H‐NMR spectroscopy

^c)^ Determined by SEC in 18 MΩ ultra‐pure water (Milli‐Q water) (0.1 m NaNO_3_, 0.025 wt% NaN_3_)

^d)^ Determined by DLS in PBS at pH 7.4

* Separate batch of non‐labeled PPz‐*g*‐PGA and PGA polymers

** Sample eluting at the limit of the column and hence *M_n_* considerably underestimated.

The bottlebrush polymers were fluorescently labeled to facilitate biological studies. In contrast to radiolabels, fluorescent tags allow visualization of the marked entity for a longer timeframe. However, several prerequisites should be fulfilled, such as a stable fluorophore and linkage point, as well as minimal loading to avoid any changes in the polymer conformation due to the conjugated tag.^[^ ^30^ ^]^ In view of these requirements, cyanine 5 (Cy5) was chosen as fluorophore and covalently linked to the bottlebrush polymers via a stable amide bond at the *N*‐terminus of the peptide chains. The polymers were labeled in their benzyl‐protected form, P2.1, P2.2, P2.3 and P3, and subsequently deprotected, P4.1, P4.2, P4.3 and P5, (Figure 1) since the *N*‐terminus of the PGA side chains was unavailable after deprotection due to the formation of a cyclic pyroglutamic acid moiety.^[^ ^37^ ^]^ The presence of the amine in P2 and its unavailability after deprotection (P4) was confirmed by a ninhydrin test. Subsequent removal of the benzyl‐protecting groups was performed with trimethylsilyl iodide (TMSI).^[^ ^37^ ^]^ TMSI was chosen in contrast to simpler acidic or basic hydrolysis since the PPz backbone is susceptible to hydrolysis at low pH,^[^ ^49^ ^]^ while racemization of the α‐carbon was observed in the ^1^H‐NMR spectroscopy upon deprotection with NaOH. The deprotection with TMSI prevented backbone degradation and minimized any observable racemization of the α‐carbon resulting in highly water‐soluble PPz‐*g*‐PGA polymers P4.1, P4.2, P4.3, and linear PGA P5.

**Figure 2:**
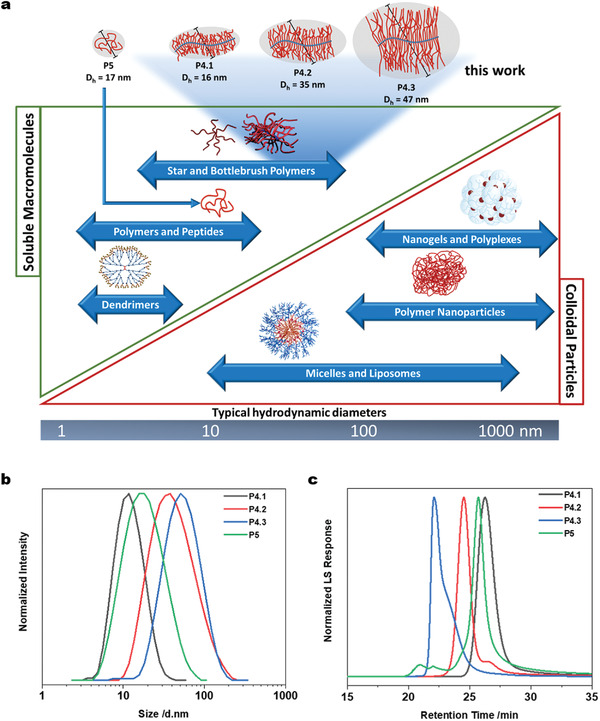
a) Overview of classical polymeric systems used as drug delivery vehicles and their typical Dh_z‐Average,_ including a schematic representation of the series of PPz‐*g*‐PGA bottlebrush polymers (P4.1–P4.3) of increasing MW and Dh_z‐Average_ as well as a linear PGA reference polymer (P5). b) Normalized intensity DLS‐plots of PPz‐*g*‐PGA bottlebrush polymers in PBS pH 7.4. c) SEC‐LS traces of PPz‐*g*‐PGA bottlebrush polymers in aqueous media.

The final polymers were characterized by NMR spectroscopy, UV/Vis‐spectroscopy, CD‐spectroscopy, aqueous SEC and dynamic light scattering (DLS), summarized in Table 1 and depicted in **Figure** **2** and Figures S8–S16, Supporting Information. As expected, SEC and DLS measurements indicated an increase in MW and hydrodynamic diameter (Dh) in accordance with the increasing length of the PGA side chains. Unfortunately, direct measurement of the Cy5‐labeled polymers was only partially possible for SEC in aqueous media and not possible for DLS due to the overlapping absorbance of the Cy5‐fluorescent tag. This was overcome by synthesizing a discrete series of polymers, analogues to P4.1, P4.2, P4.3 and P5, but without any fluorescent tag, denoted with * in Table 1. For this series, an increase in Dh from 16 nm for P4.1 up to 47 nm for P4.3 was observed. As depicted in Figure 2a, the Dh range of our bottlebrush polymers is extremely large for such individual macromolecules, thus pushing them into the size realms of more complex, dynamic self‐assembled nanostructures but with the advantage of a completely solubilized unimolecular carrier of high structural integrity. The linear PGA P5 showed a similar hydrodynamic volume to bottlebrush polymer P4.1, despite its lower number of glutamic acid units (GAU), which can be explained by the densely branched architecture (Figure 2b). The number of GAU was estimated by ^1^H‐NMR spectroscopy to give a relative number of amino acids per macromolecule and hence functional sites for future conjugation to active compounds. The same trend for the Dh can be observed in the apparent MW measured by SEC, increasing from P4.1 at 24 kDa, over P4.2 at 231 kDa, to P4.3 at 248 kDa. Although the increase in *M_n_* from P4.2 to P4.3 was smaller than expected, it may be explained by the limits of the column leading to an underestimated *M_n_* for P4.3. The linear PGA P5 was chosen as a reference for biological studies, due to its similar GAU to PGAs that have been most widely used in previous studies.^[^ ^30^, ^50^ ^]^ The aqueous SEC measurements of the Cy5‐labeled polymers (Figure 2c) show monomodal peaks for all polymers with relatively narrow Ð. The successful labelling of the polymers was again verified via SEC. Additionally, the configuration of the synthetic polypeptide side chains was determined via CD‐spectroscopy both before and after deprotection of the benzyl ester. The spectra are depicted in Figure S16, Supporting Information, and show an α‐helical configuration of the benzyl‐protected peptide side chain in 1,1,1,3,3,3‐hexafluoro‐2‐propanol, which collapses into a random coil formation of the deprotected PGA side chains in phosphate‐buffered saline (PBS) at pH 7.4, as described previously.^[^ ^30^ ^]^ The ^31^P‐NMR spectra showed the characteristic broad peak at around 0 ppm, confirming the sole and intact phosphorus species in the PPz backbone (Figures S9, S11, and S13, Supporting Information).

The degradation behavior of the bottlebrush polymers was tested using P4.2 as a model and compared to the linear analogue P5. To this end, the polymers were dissolved in sodium acetate buffer at pH 5.5 and digested with cathepsin B in the presence of ethylenediaminetetraacetic acid (EDTA) and dithiothreitol (DTT). The decrease in MW and hence degradation of the polymers was followed via aqueous SEC measurements, showing that the brush PGA polymers (P4.2) degrade similarly to the linear PGA polymer P5 (Figure S17, Supporting Information). In combination with the degradable PPz backbone, the complete degradability of the bottlebrush polymers toward biocompatible end‐products, glutamate for PGA and phosphate and ammonium salts for PPz, is therefore provided, as described in the literature for both polymers.^[^ ^30^, ^49^ ^]^

### 2.2 Cellular Uptake Studies

**Figure 3:**
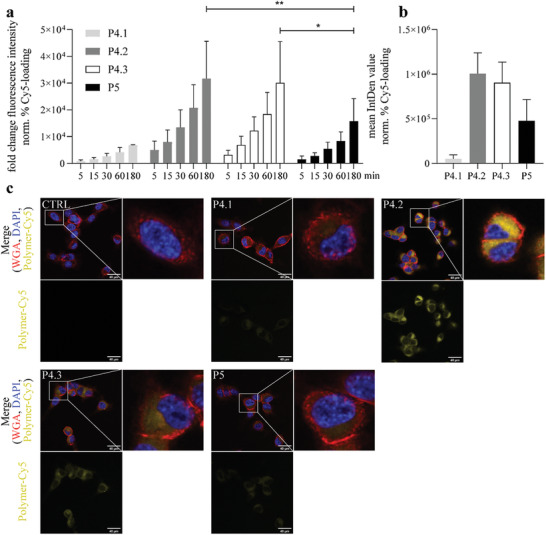
Uptake of Cy5‐labeled polymers by CT26 cells in cell culture. The fluorescence intensities were measured by a) flow cytometry after incubation for the indicated time points as well as b,c) confocal microscopy. a) Values given in the graph are the fold change of fluorescence intensity ± standard deviation (SD) compared to untreated cells of three independent experiments, normalized to the Cy5 loading of the polymers. The significance to P5 was determined using two‐way ANOVA analysis with Dunnett's multiple‐comparison test (** *p* < 0.01, * *p* < 0.05). b) Confocal microscopy was performed after incubation with 1 mg mL^−1^ of the polymers for 1 h. Three independent experiments were performed. Of each experiment, three pictures per spot were taken and the mean fluorescence (mean integrated density [IntDen] values) ± SD were analyzed by ImageJ. The fluorescence intensities were corrected to the respective Cy5 loading of the polymer. c) Representative confocal microscopy images. Polymers are indicated in yellow, nuclei (blue) and membranes (red) were co‐stained by 4′,6‐diamidino‐2‐phenylindol (DAPI) and wheat‐germ agglutinin (WGA), respectively (scale bar: 40 µm).

Murine CT26 colon carcinoma cells were incubated with the Cy5‐labeled polymers and evaluated at several time points by flow cytometry. As depicted in **Figure** **3**a, all polymers show a time‐dependent increase in cellular fluorescence, with significant differences between them. P4.1, the smallest bottlebrush polymer, had the lowest cell association and uptake, followed by the linear version P5. Interestingly, P4.2 and P4.3 had significantly higher accumulation (approximately twofold) in the cells than P5 after 180 min. A similar, albeit less exaggerated effect, has been observed by Vicent et al. for star‐branched PGA,^[^ ^30^ ^]^ attributing this to the branched architecture. This pattern of cell association and polymer internalization was subsequently confirmed by confocal microscopy (Figure 3b,c). Taking our findings and those of Vicent et al. together, it seems that both size and architecture play a role in the clearly enhanced cell uptake. PGA‐based polymers are well‐known to be effectively internalized by endocytosis.^[^ ^3^, ^51^ ^]^ Interestingly, confocal microscopy also revealed a clear and rapid cytosolic localization of the Cy5 dye, strongly indicating a lysosomal degradation of the polymer carrier and release of Cy5 into the cytosols of the cancer cells.

### 2.3 Pharmacokinetic Studies

**Figure 4:**
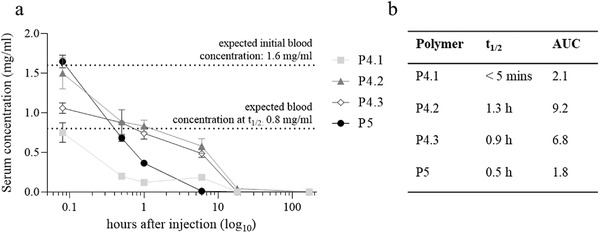
Pharmacokinetic studies in tumor‐free Balb/c mice treated with P4.1, P4.2, P4.3, or P5 (100 mg kg^−1^ i.v. dissolved in 0.9% NaCl). After 5 min, 30 min, 1 h, 6 h, 18 h and one week, blood was collected from the facial vein, followed by measurement of Cy5 fluorescence intensity in the serum. a) Values given are Cy5‐labeled polymer concentrations in the serum (mg mL^−1^) ± SD in triplicates. b) Serum t_1/2_, as well as the AUC values, were calculated in GraphPad Prism.

Tumor‐free, healthy mice were injected with 100 mg kg^−1^ Cy5‐labeled polymer intravenously (i.v.), followed by blood sampling via the facial vein at different time points (5 min to 1 week). Cy5 fluorescence was detected by a plate reader and the values were corrected for the relative fluorophore concentration of the individual polymers. Experiments were performed in both males and females, however, without significant differences between the genders. Despite a general clear biphasic behavior, the various polymers showed striking differences in their pharmacokinetics (**Figure** **4**). At the first time point (5 min), the smallest bottlebrush polymer P4.1 had distinctly lower serum levels than P4.2 and P4.3 (0.8 mg mL^−1^ versus 1.5 and 1.1 mg mL^−1^, respectively). Interestingly, although the reference polymer P5 has a similar Dh as P4.1, it showed the highest serum concentration after 5 min with 1.6 mg mL^−1^. However, for P5, a dramatic drop to 0.7 mg mL^−1^ could be observed after 30 min, which is below the levels of P4.2 and P4.3 with 0.9 mg mL^−1^ in both cases. Considering an expected initial blood concentration of 1.6 mg polymer/mL directly after the application, the low polymer levels at the first measurement point indicate that P4.1 has a faster alpha phase clearance with a serum *t*_1/2_ below 5 min (Figure 4b). Additionally, the assumption of a very fast excretion of P4.1 is in agreement with the observation of strongly blue‐colored urine of the treated animals after 5 min of injection (data not shown). The longest *t*_1/2_ were found for P4.2 (1.3 h) and P4.3 (0.9 h). Thus, both polymers also exhibited the largest area under the curve (AUC) of 8.2 and 6.8, respectively, indicating a long beta phase and, thus, slow excretion of the polymers. At the 5 min timepoint the observed plasma levels of P4.3 were lower than those of P4.2, however data points in the first few minutes always must be considered with caution due to the highly dynamic phase soon after injection.

Although P5 showed the highest concentration in the serum after 5 min, it displayed a *t*_1/2_ of only 0.5 h. After 18 h, no Cy5 fluorescence for any polymer could be detected in the serum. Regarding the behavior of P5 and P4.1, the effect of the polymer architecture could play a decisive role, as their hydrodynamic diameter (measured either by SEC or by DLS) was rather similar (DLS measurement, 16 versus 17 nm as indicated in Figure 2a). Macromolecules with similar Stokes–Einstein radius but differences in polymer architecture (branched versus linear) can strongly differ in terms of renal filtration^[^ ^52^ ^]^ and biodistribution.^[^ ^4^ ^]^ When comparing P5 and P4.1, apparently the short PGA brushes attached to PPz of P4.1 (average two times 2–3 GA residues per repeating unit) result in a rather rod‐like molecule which is shorter than P5, however, its elongated diameter would be sufficiently small to pass the pores. For polymers P4.2 and P4.3 the PGA side chains consist of on average *N*_SC_ of 20 (P4.2) and 87 (P4.3) GAU, respectively. In a simulation study, the chain length of PGA was estimated to be in the range of 3 nm (for six GAU).^[^ ^53^ ^]^ With an average chain length of 20 GAU in the P4.2 brush, this would result in a theoretical rod diameter of >6 nm and hence significantly decreased glomerular filtration would be expected (vide infra).

### 2.4 Organ Distribution and Tumor Accumulation Measurement by Fluorescence and Bioluminescence Imaging In Vivo

**Figure 5:**
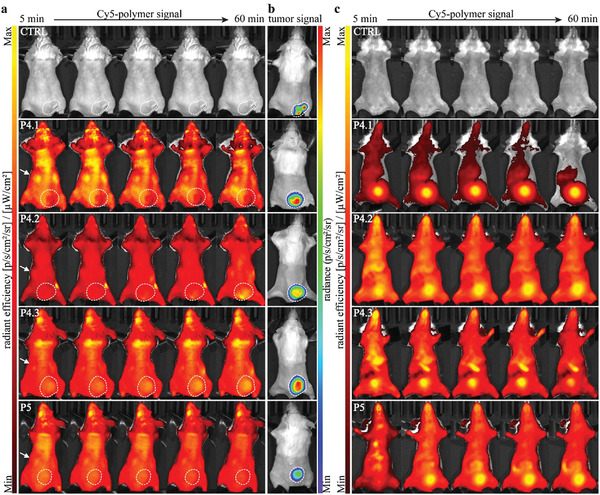
Biodistribution studies of the polymers in CT26_luc_ tumor‐bearing mice on a low fluorescent diet (prone and supine position). On day 10 and 11, different sets of mice were treated with the indicated polymers (100 mg kg^−1^ i.v. dissolved in 0.9% NaCl), followed by IVIS measurement in a supine or prone position for 1 h, respectively. a) Representative images of Cy5 radiance efficiency of one mouse in prone position per polymer, 5, 15, 30, 45 and 60 min after injection. b) Associated BLI images of tumor signal on the day of polymer injection. Arrows indicate the kidneys and tumors are encircled. c) Representative images of Cy5‐radiance efficiency of one mouse in supine position per polymer, 5, 15, 30, 45 and 60 min after injection.

To further study the biodistribution and tumor accumulation, the polymers were tracked by 2D epifluorescence imaging (FLI) in (luciferase‐transfected) CT26_luc_ tumor‐bearing mice (on a low‐fluorescent diet). Different sets of animals were measured in a prone or supine position, flipped (from prone to supine and vice versa) and measured in the other position, as detailed in Figure S18, Supporting Information. This was done to evaluate the biodistribution in all possible organs in all animals in both prone and supine positions. Tumor location and growth were visualized by firefly luciferase‐based bioluminescence imaging (BLI) on different days, as shown in Figures S19 and S20, Supporting Information. On days 10 and 11 of tumor growth, the Cy5‐polymers were injected i.v. at a dose of 100 mg kg^−1^ into different sets of mice for supine and prone measurement, respectively. The biodistribution of Cy5‐labeled polymers was tracked in real‐time by FLI over a 1 h period to study the initial dynamic phase of organ distribution. **Figure** **5** shows one representative mouse per polymer and an untreated control mouse in the prone and supine position for visualizing polymer presence via FLI signal (Figures 5a and 5c, respectively) and tumor location via BLI signal (Figure 5b). The fluorescence could be easily detected in all polymer‐treated animals compared to the untreated control animal, indicating a good background‐to‐signal ratio. All animals treated with bottlebrush polymers showed kidney (Figure 5a and Figure S21, Supporting Information) and bladder signals (Figure 5b and Figure S21, Supporting Information) within 5–10 min after injection. Out of the three bottlebrush polymers, P4.1 displayed the highest signal in the bladder and was also the fastest to reach the bladder within 5–10 min of injection, in close agreement with the pharmacokinetic data in which P4.1 had the fasted excretion (see Figure 4). The organ distribution behavior for the linear P5 polymer was similar to that of P4.1. In addition, several mice already showed indications for a strong liver‐associated signal, especially in the case of P4.2 and P4.3. As shown in Figure 5, and Figures S21 and S24, Supporting Information, there was an indication for polymer accumulation in the tumor in some of the P4.2 and P4.3‐treated animals, which could already indicate the onset of the enhanced permeability and retention (EPR) effect.^[^ ^54^ ^]^

**Figure 6:**
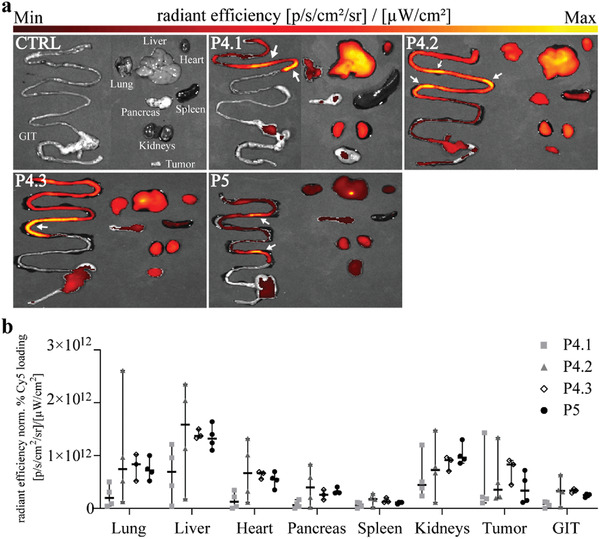
Ex vivo Cy5 fluorescence intensity measurements of tumor and organs. After 1 h of IVIS Spectrum imaging, mice were sacrificed and a) Cy5 radiance efficiency of tumor as well as organs (lung, liver, heart, pancreas, spleen, and kidney) measured (representative images of one mouse per polymer group each, order of the organs is always the same). Arrows indicate feces. b) Quantification of Cy5‐radiance efficiency of tumor as well as organs (*n* = 4). Fluorescence intensities were corrected to the respective Cy5 loading of the polymers (GIT = gastrointestinal track).

The animals were sacrificed after 1 h, and the organs were collected for ex vivo epifluorescence measurements (**Figure** **6**a) to gain more insight into the tissue distribution and validation. As already indicated by the real‐time data of the living mice, strong signals were seen in the kidneys and the liver, with particular brightness in the gallbladder. Moreover, the (small) intestine gave a strong signal with a special brightness associated to the feces (indicated by the arrows in Figure 6a). To allow the comparison of the brightness between the polymers and to consider their different Cy5‐loading, the regions of interest (ROIs) of the pictures were collected and corrected for the Cy5‐loading. As shown in Figure 6b, the liver and kidney were the organs with the highest Cy5 fluorescence, followed by the tumor tissue and the lung. In good agreement with their longer *t*_1/2_, the larger bottlebrush polymers P4.2 and P4.3 had higher tissue levels than the other two polymers.^[^ ^55^ ^]^ In addition, serum and urine were sampled after 1 h (Figure S25, Supporting Information), and Cy5 fluorescence analyzed. In line with the pharmacokinetic data, after 1 h the highest fold change of Cy5 fluorescence intensity in serum could be measured in P4.2‐treated animals, whereas the lowest one was detected in P4.1‐treated mice (Figure S25a, Supporting Information). Interestingly, in the urine, the highest fold change of Cy5 fluorescence intensity was quantified in P4.1‐ and P5‐treated mice, strengthening our conclusion that those polymers excreted the fastest (Figure S25b, Supporting Information).

### 2.5 Cellular Distribution of the Cy5‐Signals by Histological Evaluation

**Figure 7:**
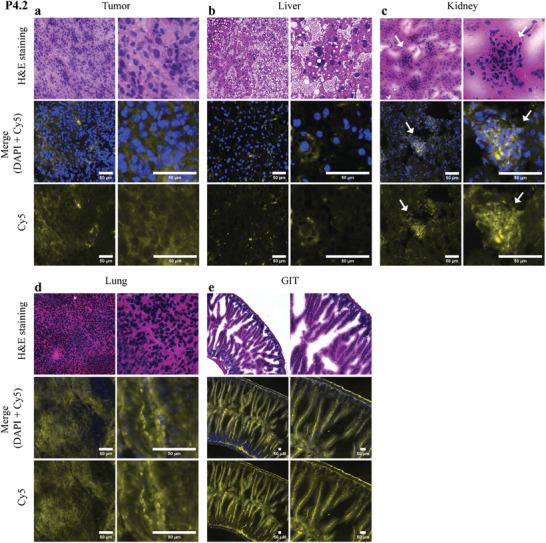
Representative fluorescence microscopy images of tissues collected from P4.2‐treated mice after the IVIS measurements. Tissues were OCT‐embedded and snap‐frozen, followed by cutting and histological staining (H&E or DAPI). Pictures were taken by a fully automated 12 slide stage (TissueFAXS, TissueGnostics) on a Zeiss observer microscope (objective magnification 20× and 63×; scale bar: 50 µm; yellow (polymer) and blue (nucleus)). a) Tumor, b) liver, c) kidney, d) lung, and e) GIT. Arrows indicate glomeruli.

Following the 1 h ex vivo IVIS measurements, the collected tumors and organs were snap‐frozen in an optimal cutting temperature (OCT) medium and cryosectioned into 5 µm slices. Consecutive sections of each mouse were either analyzed for the polymer‐associated Cy5‐signal or stained with hematoxylin and eosin (H&E) to allow histological localization of these signals (**Figure** **7** and Figures S26–S29, Supporting Information). In general, due to the extended thickness and the cryoembedding, the tissue architecture was not fully retained, especially in the lung samples. However, the tissue distribution of the polymers was still clearly visible. Thus, in good agreement with their swift elimination (blue urine after 5 min, lower fluorescence signals in IVIS measurements), P4.1 and P5 were already excreted below the detection limit in most organs at the time of sample collection (Figures S27 and S29, Supporting Information). Also, no signals were observable in the tumors of these polymers, indicating that the alpha‐phase of these polymers might be too pronounced for efficient tumor enrichment via the EPR effect (Figures S27a and S29a, Supporting Information). Moreover, especially in case of P5, some polymer aggregates were visible in the capillary bed of the lung as well as in the liver (Figures S29d and S29b, Supporting Information). In contrast, visibly higher Cy5‐associated fluorescence could be observed in tumor and organs of P4.2‐ and P4.3‐treated animals (Figure 7 and Figure S28, Supporting Information) which is again in good agreement with the PK and imaging data shown above. Especially with P4.2, a clear Cy5‐signal could be observed in the tumor tissue (Figure 7a). In general, a very even and homogenous distribution of the fluorescence signal throughout the tissues was visible for these two polymers, indicating an association of the strong signals with the higher polymer content in the blood. No aggregates of P4.2 and P4.3 were detected apart from the kidney, where some accumulation hotspots in the glomeruli were found (Figure 7c and Figure S28c, Supporting Information). Interestingly, microvilli of the gastrointestinal tract (GIT) exhibited strong Cy5‐signal in P4.2 and P4.3‐treated animals (Figure 7e and Figure S28e, Supporting Information). This could possibly be explained by the naturally occurring strong blood perfusion of this tissue. Nevertheless, based on the very intense Cy5‐signal of the feces, also reabsorption after hepatic excretion cannot be ruled out. In contrast to the confocal microscopy images, it is not possible to normalize the histological images to account for the differences in the Cy5‐loading of the polymers. Consequently, the lack of detection in case of P4.1 and P5 could be due to the lower loading of these polymers.

## 3 Conclusion

We described the design and synthesis of novel, fully biodegradable PPz‐*g‐*PGA bottlebrush polymers with high MW and, thus, hydrodynamic diameters in the Dh ≈ 16–47 nm range. The innovative methodology offers high control over the macromolecular architecture and delivers well‐defined bottlebrush polymers with ultra‐high valency. Such polymers are of high interest as unimolecular nanocarriers due to their advanced architecture in the nanometer range, alongside excellent aqueous solubility and complete biodegradability to endogenous small molecules. In vitro studies showed a direct correlation between the macromolecular dimensions of the polymers and their accumulation in CT26 murine colon cancer cells, with increased cell‐uptake for the larger bottlebrush polymers. Pharmacokinetic analysis revealed extended plasma *t*_1/2_ for the larger bottlebrush polymers with a larger area under the curve (AUC), indicating a long beta phase and, thus, slow excretion of the polymers compared to the commonly used linear PGA. Despite a similar Stokes–Einstein radius and number of amino acid units, real‐time epifluorescence measurements in mice showed that P4.1 (Dh ≈ 16 nm) had much faster renal filtration than P5 (Dh ≈ 17 nm), suggesting a considerable impact of the molecular architecture, and not just size. Meanwhile, the urine levels of P4.2 and P4.3 (Dh ≈ 35 and 47 nm) were much lower, directly resulting in higher organ and tumor levels in the treated animals. Moreover, they also indicated that the larger polymers were enriched in the glomeruli region of the kidneys, underlining the observation that these polymers are above the renal clearance limit. The real‐time epifluorescence measurements also showed considerably enhanced organ retention for the larger bottlebrush polymers (P4.2 and P4.3) and indicated increased tumor accumulation in CT26‐tumor‐bearing mice (especially for P4.2), an observation confirmed by subsequent histological analysis of the collected tissues. Moreover, a first onset of the EPR effect could be observed in some mice, underlining their potential as anticancer nanomedicines. In summary, this work introduces a robust, controlled synthesis route to PPz‐*g*‐PGA bottlebrushes which overcome some of the main limitations of current PGA‐based carriers and, in doing so, underlines the importance of macromolecular architecture on the ultimate in vivo performance of nanomedicines. Future work will aim to further study the effect of polymer architecture on different cell types and tumor models.

## 4 Experimental Section

### Materials

Chemicals were purchased from different vendors and used as purchased. 2,2′‐(ethylendioxy)‐bisethylamine, tetrafluoroboric acid diethyl ether complex (HBF_4_.Et_2_O), and dodecylamine were bought from Aldrich, 5‐benzyl *L*‐glutamate NCA from Fluorochem and cyanine 5‐NHS ester from Lumiprobe. Phosphorus trichloride (PCl_3_), 1,1,1,3,3,3‐hexafluoro‐2‐propanol (HFIP), triethylamine (Et_3_N), di‐tert‐butyl dicarbonate (Boc_2_O) and Na_2_S_2_O_3_ were purchased from Merck, LiN[Si(CH_3_)_3_]_2_, 4‐(diphenylphosphino)styrene, hexachloroethane, TMSI, DTT, EDTA, sodium acetate, SO_2_Cl_2_ and cathepsin B from human liver (in 50 mm sodium acetate buffer, 1 mm EDTA, pH 5) from Sigma, ninhydrin from Alfa Aesar and MgSO_4_, NaHCO_3_ and molecular sieves (3 Å) from VWR. Solvents were used as purchased unless specified otherwise. DMF, Et_2_O, ethanol (EtOH) and methanol (MeOH) were purchased from VWR, anhydrous dichloromethane (DCM) and anhydrous tetrahydrofuran (THF) from Alfa Aesar, Et_2_O from TCI, and DCM as well as acetic acid (AcOH) from Chem Lab. The deuterated solvents chloroform (CDCl_3_), water (D_2_O) and dimethylsulfoxide (DMSO‐d_6_) were bought from Eurisotop. Dialysis was carried out using Spectrum Spectra/Por RC membrane tubing with different molecular weight cut offs (MWCOs), EtOH used as a solvent was recycled by distillation. Ultrafiltration was performed with Vivaspin 6 centrifugal concentrators with a MWCO of 30 kDa. 18 MΩ ultra‐pure water (Milli‐Q water) was obtained from a Millipore device with a Millipak express 40 filter (0.22 µm pore size). Et_3_N was purchased from Merck and distilled and dried over molecular sieves (3 Å) prior to use. DCM was purified via a solvent purification column (MBraun SPS compact). Air‐ and moister‐sensitive syntheses were carried out under an inert atmosphere in an argon‐filled glovebox (MBRAUN).

For the NMR measurements a Bruker Avance III 300 MHz spectrometer as well as a Bruker Avance 500 MHz spectrometer were used. On the latter, the ^31^P‐NMR measurements of P2.2, P2.3, P4.2, and P4.3 were performed. Due to their high MW, the measurements were carried out with increased polymer concentration at 330K and excitation via the protons of the linker moiety. SEC in organic media was performed on a Viscothek GPCmax instrument using a PFG column from Polymer Standard Service GmbH (PSS) (Mainz, Germany) (300 mm × 8 mm, 5 µm particle size). The samples were filtered through 0.2 µm PTFE syringe filters prior to injection and eluted with DMF containing 10 mm LiBr as the mobile phase at a flow rate of 0.75 mL min^−1^ at 60 °C. The molecular weights were measured using a multiple detector calibration of the light scattering, refractive index, and viscosity detectors calibrated with a polystyrene standard from Polymer Standard Service GmbH (PSS). SEC in aqueous media was performed on an Agilent Technologies 1260 Infinity II system equipped with a Shodex OHpak LB‐802.5 (300 mm × 8 mm, 6 µm particle size) and a Shodex OHpak LB‐804 (300 mm × 8 mm, 10 µm particle size) column. Samples were filtered through 0.2 µm nylon syringe filters prior to injection, eluted with Milli‐Q water with 0.1 m NaNO_3_ and 0.025 wt% NaN_3_ at a flow rate of 0.5 mL min^−1^ and detected via a UV–Vis detector from Agilent, a refractive index detector RI‐501 from Shodex and a TREOS II light scattering detector from Wyatt technology. Results were analyzed with ASTRA 7.3.2 from Wyatt with a d*n*/d*c* value of 0.1675 for PGA in H_2_O. DLS measurements were performed on a Malvern Zetasizer Nano ZSP at 25 °C at a fixed scattering angle of 173°. The sample solutions (0.2 mg mL^−1^) were prepared in PBS pH 7.4, sonicated for 10 min, and filtered through 0.2 µm nylon syringe filters prior to the measurement. Circular dichroism spectrometry was performed on a J‐810‐150S spectropolarimeter from Jasco with a PTC‐423S temperature control system from Jasco and 1 mm quartz cuvettes from Helma Analytics. Measurement settings were set to a measure range from 240–190 nm with 0.5 nm data intervals and a scanning speed of 20 nm min^‐1^. Samples were analyzed with 3 accumulations and the data with Spectra Manager, version 2.14.02. UV–Vis spectra were recorded in PBS pH 7.4 with 1% DMSO on a SpectraMax M2e Multi‐Mode Microplate Reader with applied wavelengths of 350–780 nm. Samples were lyophilized with an Alpha 1–2 LDplus freeze‐drying system from Christ.

### Synthesis of Trichloro‐*N*‐(trimethylsilyl)‐phosphoranimine

The PPz backbone monomer was synthesized based on adapted literature procedures.^[^ ^56^ ^]^ Briefly, 25.08 g (0.15 mol) of LiN[Si(CH_3_)_3_]_2_ was dissolved in Et_2_O and cooled to 0 °C in an ice bath. Then, 13.11 mL (0.15 mol) of PCl_3_ were added slowly over the course of ≈1 h. After stirring for 1 h at 0 °C, 12.11 mL (0.15 mol) SO_2_Cl_2_ were added dropwise over the course of ≈1 h and stirred again for 1 h at 0 °C. Subsequently, the reaction mixture was allowed to warm to room temperature (r.t.), filtered through dry Celite, and the solvent was evaporated in a water bath at r.t. Finally, vacuum distillation was performed at 1–5 mbar and 40 °C resulting in Cl_3_PNSi(CH_3_)_3_ as a clear and colorless liquid. The product was transferred into a glove box and stored in the freezer.

Yield: 22.01 g (65%); ^1^H‐NMR (300 MHz, CDCl_3_, δ): 0.17 ppm (d, 9H); ^31^P‐NMR (121 MHz, CDCl_3_, δ): ‐54.56 ppm

### Synthesis of *tert*‐butyl *N*‐(2‐[2‐(2‐aminoethoxy)ethoxy]ethyl) Carbamate

Synthesis of the PPz macrosubstituent was performed according to adapted literature procedure.^[^ ^57^ ^]^ Briefly, 26.7 mL (0.183 mol, 1 equiv.) 2,2′‐(ethylenedioxy)‐bisethylamine was dissolved in 200 mL DCM at 0 °C under Ar. Then, 4.04 g (0.0185 mol, 0.1 equiv.) di‐*tert*‐butyldicarbonate was dissolved in 200 mL anhydrous DCM and gradually added to the cooled reaction solution over the course of 6 h. The reaction solution was then allowed to warm to r.t. and stirred overnight. The solvent was evaporated, the product taken up with 200 mL deionized water (diH_2_O) and extracted 5‐times with 150 mL DCM. The combined organic phase was washed with 200 mL diH_2_O, dried over MgSO_4_, and evaporated at reduced pressure yielding a clear yellowish product.

Yield: 4.43 g (96%); ^1^H‐NMR (300 MHz, CDCl_3_, δ): 1.33 (s, 9H), 2.76 (br, 2H), 3.19 (br, 2H), 3.38‐3.45 (m, 4H), 3.50 ppm (br, 4H)

### Synthesis of Poly(*tert*‐butyl *N*‐(2‐[2‐(2‐aminoethoxy)ethoxy]ethyl) carbamate)phosphazene

The PPz was synthesized in the glove box at r.t. based on typical literature procedures.^[^ ^58^ ^]^ Briefly, 4‐(diphenylphosphino)styrene (0.01 g, 0.03 mmol, 1 equiv.) was dissolved in little anhydrous DCM and combined with C_2_Cl_6_ (0.009 g, 0.04 mmol, 1.1 equiv.), dissolved in around 1 mL anhydrous DCM as well. The solution was stirred overnight resulting in the chlorinated initiator species. For polymerization, Cl_3_PNSi(CH_3_)_3_ (584 mg, 2.60 mmol, 75 equiv. for *n* = 75) as the monomer was dissolved in ≈1–2 mL anhydrous DCM, added to the initiator solution and stirred for around 24 h. The macrosubstitution of the chlorine substituents was performed by dissolving *tert*‐butyl *N*‐(2‐[2‐(2‐aminoethoxy)ethoxy]ethyl) carbamate (1.615 g, 6.53 mmol, 187.5 equiv.) in around 80 mL THF. Then, 0.91 mL (6.53 mmol, 187.5 equiv.) Et_3_N were added to the substituent solution and the polymer solution was dropped in gradually resulting in a cloudy, white solution. The reaction was stirred for 24 h, removed from the glove box, and filtered through filter paper. THF was evaporated and the polymer purified by dialysis against diH_2_O for 2 h, following 22 h in EtOH with a MWCO of 3.5 kDa. Finally, the solvent was removed under reduced pressure yielding a highly viscous yellowish polymer.

Yield: 995.3 mg (71%); ^1^H‐NMR (300 MHz, CDCl_3_, δ): 1.43 (s, 9H), 3.01 (br, 2H), 3.28 (br, 2H), 3.51 (br, 4H), 3.58 (br, 4H), 5.50 ppm (br, 1H); ^31^P‐NMR (121 MHz, CDCl_3_, δ): 2.26, 4.75, 11.03, 11.74 ppm

### Synthesis of Poly(2,2′‐(ethane‐1,2‐diylbis(oxy))bis(ethan‐1‐amine))phosphazene BF_4_ Salt (P1, macroinitiator)

The deprotection of the BOC‐protected poly(tert‐butyl N‐(2‐[2‐(2‐aminoethoxy)ethoxy]ethyl) carbamate)phosphazene and formation of the macroinitiator P1 was performed according to adapted literature procedure.^[^ ^29^, ^30^ ^]^ Briefly, the polymer (304.4 mg, 0.5641 mmol, 1 equiv.) was dissolved in 50 mL anhydrous DCM and HBF_4_.Et_2_O (460.6 µL, 3.3847 mmol, 6 equiv.) was added slowly under vigorous stirring. The solution turned slightly brownish and a white precipitate was formed. After 5–10 min the solution was filtered through a filter paper and the precipitate washed 5‐times with 50 mL cold Et_2_O. Afterward, the precipitate was re‐dissolved from the filter paper with MeOH, the solvent was evaporated and the product dried under vacuum yielding a clear to white solid.

Yield: 231.5 mg (80 %); ^1^H‐NMR (300 MHz, D_2_O, δ): 3.19 (br, 4H), 3.50‐3.82 ppm (m, 8H); ^31^P‐NMR (121 MHz, D_2_O, δ): 4.29 ppm;^19^F‐NMR (470.59 MHz, D_2_O, δ): −149.97 ppm

### Synthesis of Dodecylammonium BF_4_ Salt (Initiator)

The synthesis of the initiator was performed according to adapted literature procedures.^[^ ^29^, ^30^ ^]^ Dodecylamine (158.8.2 mg, 1.0262 mmol) was dissolved in 50 mL anhydrous DCM and HBF_4_. Et_2_O (349.1 µL, 2.5654 mmol, 2.5 equiv.) added under vigorous stirring upon which the solution turned slightly orange. A white precipitate formed almost immediately and the reaction was allowed to proceed for 5–10 min. The precipitate was filtered off through a filter paper and washed 5‐times with 50 mL cold Et_2_O. Finally, the precipitate was re‐dissolved with little MeOH, the solvent was evaporated and the product dried under vacuum.

Yield: 128 mg (46 %); ^1^H‐NMR (300 MHz, DMSO‐d_6_, δ): 0.76‐0.93 (m, 3H), 1.25 (s, 18H), 1.50 (br, 2H), 2.76 (t, 2H), 7.57 ppm (s, 3H);^19^F‐NMR (470.59 MHz, DMSO‐d_6_, δ): ‐148.26 ppm

### Polymerization of PPz‐*g*‐PBLGA and PBLGA (P2 and P3)

Polymerization of the PBLGA, the linear analogue as well as the grafts on the bottlebrush polymers, was performed in an argon‐filled glove box according to adapted literature procedures.^[^ ^29^, ^30^ ^]^ NCA (1.517 g, 5.4757 mmol, 20 equiv., for *m* = 10, see **Table** **2**) was dissolved in 14.41 mL DMF, previously dried over molecular sieve (3 Å) and bubbled through with N_2_, to achieve a 0.38 m solution of the NCA and added to the initiator (141.0 mg, 0.2738 mmol). The solution was stirred in the glove box for 4 days and, subsequently, precipitated in cold Et_2_O, 10‐times the volume of DMF used for the polymerization. After centrifugation (4000 rpm, r.t., 10 min), the precipitate was collected and dried under vacuum yielding a yellowish to white polymer.

**Table 2:** Summarized amounts of initiator, NCA‐monomer, and solvent used for the polymerization of P2 and P3 along their respective yields.

Polymer	*m*_NCA_ [g]	*n*_NCA_ [mmol]	*m*_Init._ [mg]	*n*_Init._ [mmol]	*V*_DMF_ [mL]	Yield [mg]	Yield [%]
P2.1	1.517	5.4757	141.0	0.2738	14.41	284.7	22
P2.2	1.415	5.1068	26.3	0.0511	13.44	274.0	24
P2.3	1.452	5.2427	13.5	0.0262	13.80	553.9	48
P3	1.582	5.7107	2.6	0.0095	15.03	800.2	64

Exemplary NMR‐Spectra of P2.1: ^1^H‐NMR (300 MHz, DMSO‐d_6_, δ): 1.64‐2.12 (br, 2H), 2.18‐2.51 (br, 2H), 4.99 (s, 2H), 7.26 ppm (s, 5H); ^31^P‐NMR (121 MHz, DMSO‐d_6_, δ): 4.26, 10.78 ppm.

### Conjugation of Cy5‐NHS Ester via the *N*‐terminus to P2.1, P2.2, P2.3, and P3

The polymers (P2.1, P2.2, P2.3, or P3) (200 mg) were dissolved in 3 mL DMF, previously dried over molecular sieve (3 Å) and bubbled through with N_2_, in the glove box and Et_3_N (0.2 mL) was added. Cyanine 5‐NHS ester (1 mg, 0.0015 mmol) was weighed in for a loading of 0.5 mg/100 mg polymer and added to the polymer solution via 2‐times 0.5 mL dry DMF. The mixture was stirred in the glove box in the dark for 24 h and, subsequently, precipitated in 50 mL cold Et_2_O. After centrifugation (4000 rpm, r.t., 10 min), the precipitate was collected, dried under air in the dark and directly used for further synthesis.

### Deprotection of the Benzyl Ester via TMSI (P4 and P5)

The deprotection of the benzyl group was performed according to adapted literature procedures.^[^ ^37^ ^]^ Briefly, the polymers (P2.1, P2.2, P2.3, or P3) (≈200 mg) were transferred into an argon‐filled glove box, dissolved in 5 mL anhydrous DCM, and reacted with TMSI (6 equiv. per glutamic acid). The reaction was stirred in the glove box at r.t. for 24 h, the DCM subsequently evaporated, and the residue suspended in 5 mL saturated NaHCO_3_ solution and 5 mL Milli‐Q water. Upon addition of a minimal amount of Na_2_S_2_O_3_, the solution turned white (blue in case of Cy5 labelling) and was stirred for 24 h. The sample was then washed 3‐times with 5 mL cold Et_2_O, dialyzed against water for 48 h (P2.1, P2.2, P2.3 with 6–8 kDa MWCO, and P3 with 3.5 kDa MWCO) and further purified by ultrafiltration with Vivaspin 6 centrifugal concentrators (30 kDa MWCO). Freeze‐drying yielded a fluffy, white polymer (fluffy, blue in case of Cy5 labelling).

P4.1: Yield: 45%; ^1^H‐NMR (300 MHz, D_2_O, δ): 1.74‐2.14 (br, 4.9H), 2.15‐2.57 (br, 5.5H), 2.86‐3.23 (br, 2.3H), 3.23‐3.47 (br, 2.5H), 3.47‐3.81 (br, 8H), 4.30 ppm (s, 2.4H); ^31^P‐NMR (121 MHz, D_2_O, δ): 4.26, 11.03, 19.88 ppm

P4.2: Yield: 63%; ^1^H‐NMR (300 MHz, D_2_O, δ): 1.71‐2.13 (br, 38H), 2.14‐2.60 (br, 40.7H), 3.21‐3.85 (br, 12H), 4.29 ppm (s, 19.3H); ^31^P‐NMR (121 MHz, D_2_O, δ): 4.12, 11.59, 20.63 ppm

P4.3: Yield: 61%; ^1^H‐NMR (300 MHz, D_2_O, δ): 1.62‐2.12 (br, 191.9H), 2.13‐2.60 (br, 189.3H), 3.45‐3.78 (br, 8H), 4.30 ppm (s, 87.2H); ^31^P‐NMR (121 MHz, D_2_O, δ): 2.99, 11.24 ppm

P3: Yield: 72%; ^1^H‐NMR (300 MHz, D_2_O, δ): 1.24 (s, 18H), 1.67‐2.11 (m, 470.2H), 2.27 (br, 448.8H), 4.30 ppm (s, 224.3H)

### Cathepsin B Degradation Studies

The bottlebrush polymer P4.2 and its linear analogue P5 were degraded in the presence of cathepsin B according to adapted literature procedure,^[^ ^30^, ^59^ ^]^ and the degradation process was observed by aqueous SEC. The polymer (9 mg) was dissolved in 2.6 mL sodium acetate buffer (20 mm, pH 5.5) and 0.3 ml of a solution of 2 mm EDTA and 5 mm DTT. 10 µg cathepsin B in 100 µL sodium acetate buffer (20 mm, pH 5.5) was added to the mixture, resulting in a 3 mg mL^−1^ solution of the polymer, and a SEC chromatogram was recorded (*t*_0_). The sample solution was incubated at 37 °C and 100 µL aliquots were injected into the aqueous SEC system and measured at each time point.

### Cell Culture

The murine CT26 colon carcinoma cells were purchased from American Tissue Culture Collection (ATCC). The generation of CT26_luc_ cells had been described by Groza et al., 2018^[^ ^60^ ^]^ (luciferase expression was confirmed using GloMax Navigator System [GM2010, Promega]). Both cell clones were cultivated in Dulbecco's modified eagle's medium/F12 medium (1:1 from Sigma) supplemented with 10% fetal bovine serum (FBS; Life Technologies) and 1% *L*‐glutamine (Sigma‐Aldrich) and were maintained under standard cell culture conditions at 37 °C in a humidified atmosphere at 5% CO_2_.

### Flow Cytometry

5 × 10^5^ cells/mL CT26 cells were seeded in 12‐well plates and allowed to recover for 24 h at 37 °C with 5% CO_2_ overnight. Subsequently, the cells were treated with 1 mg mL^−1^ of the polymers (diluted in phenol red‐free RPMI1640 medium [Sigma‐Aldrich] containing 10% FBS) or solvent for 3 h, 1 h, 30 min, 15 min and 5 min, followed by trypsinization and two washing steps with ice‐cold PBS. Finally, the cell pellets were re‐suspended in ice‐cold FACS‐PBS. The fluorescence intensity was measured by flow cytometry using a BD LSRFortessaTM X‐20 cell analyzer (Becton Dickinson, Palo Alto, CA, USA). For these experiments, in total 3 × 10^4^ cells per sample were evaluated. The results were analyzed and quantified using BD FACSDivaTM software.

### Confocal Microscopy

CT26 cells were seeded on spot slides (Science Services) at a cell concentration of 8 × 10^4^ cells/mL (50 µL per spot). The cells were allowed to recover at 37 °C with 5% CO_2_ overnight. The next day, the cells were treated with 1 mg mL^−1^ of the test polymers (diluted in phenol red‐free RPMI 1640 medium containing 10% FBS) and incubated at 37 °C with 5% CO_2_ for 1 h. The cells were washed 3‐times with PBS and fixed with 4% paraformaldehyde (PFA) at r.t. for 30 min. After three PBS washing steps, the cells were incubated with PBS containing DAPI (2.5 µg mL^−1^) and WGA (1:500, Vector Laboratories) for 15 min at r.t., followed by three PBS washing steps as well as ddH_2_O and embedding with Vectashield (Vectashield Antifade Mounting Media, Vector Laboratories). Subsequently, confocal microscopy was performed on a Zeiss LSM 700 (Carl Zeiss AG), equipped with 405, 488, 555 and 639 nm solid state laser diodes, using a Plan‐NeoFluar 40×/NA 1.3/Oil lens. The pinhole size was set to 1 AU. Line average of 2 was applied to all channels. In total, 3 pictures per spot were obtained and quantified by the open source software Fiji.

### Animals

All experiments were approved by the Ethics Committee for the Care and Use of Laboratory Animals at the Medical University of Vienna and the University of Vienna (BMBWF‐66.009/0140‐II/3b/2011, BMBFW‐66.006/0027‐WF/V/3b/2014, BMBWF‐66.009/0157‐V/3b/2019) and performed according to the guidelines from the Austrian Animal Science Association and from the Federation of European Laboratory Animal Science Associations (FELASA), the U.S. Public Health Service Policy on Human Care and Use of Laboratory Animals as well as the United Kingdom Coordinating Committee on Cancer Prevention Research's Guidelines for the Welfare of Animals in Experimental Neoplasia.

For the pharmacokinetic studies 8‐12‐week‐old Balb/c mice were purchased from Harlan (Italy). For the IVIS imaging of tumor as well as organ distribution, 6‐week‐old Balb/c was purchased from Janvier Labs (Le Genest‐Saint‐Isle, France). All animals were kept in a pathogen‐free environment with a 12 h light–dark cycle with ad libitum access to food and water. In case of the IVIS experiments, food supply was changed to a low‐fluorescent diet (E15710‐047 EF AIN76A Purified diet, 10 mm Sterilis. 25 kGy, Sniff Spezialdiäten GmbH Ferd.‐Gabr.‐Weg 16 D‐59494 Soest) for six weeks prior to the experiments. This is necessary as tissue autofluorescence and ingested food (chlorophyll containing diet) would overlap with the Cy5 fluorescence. As a side effect of the fiber‐free diet, slight signs of macrovesicular fatty liver could be observed in H&E stains. However, this was not expected to impact the biodistribution pattern observed in the IVIS measurements. Welfare of the animals was monitored daily (e.g., body weight, fatigue, ragged coat, and food and fluid consumption). Tumor growth and possible side effects of drug treatment were evaluated every working day by recording the tumor size by caliper measurement. Tumor volumes (mm^3^) were calculated using the formula: (length × width^2^)/2. Collected data was evaluated using GraphPad Prism 8 software.

### Pharmacokinetic Studies

Female and male mice (*n* = 3 per treatment) were treated with the polymers (100 mg kg^−1^ i.v. dissolved in 0.9% NaCl). After 5 min, 30 min, 1 h, 6 h, 18 h and 1 week, blood was collected via the facial vein. Serum was isolated of the collected blood samples by centrifugation at 900 g for 10 min (r.t.) for two times. Finally, the serum samples were diluted 1:20 with ddH_2_0 and the Cy5 fluorescence intensity was measured by a fluorescence plate reader (TECAN, Infinite 200 PRO). The Cy5 concentrations were calculated using a standard curve.

### Optical Imaging Based Investigation of Organ Distribution in Tumor‐Bearing Mice

The in vivo imaging was performed using an IVIS Spectrum CT imaging system (PerkinElmer, Waltham, Ma 0 2451, USA). Figure S18, Supporting Information, shows the overall workflow of the steps from cell harvesting and tumor implantation to organ analysis.

5 × 10^5^ CT26_luc_ cells were injected subcutaneously (s.c.) into the right flank of female Balb/c mice (*n* = 4 mice per group). Cell count was determined using MACSQuant Analyzer 10 (Miltenyi Biotech, Bergisch Gladbach, Germany). Tumor growth was measured using BLI on days 1, 4, and 10 (supine group) or 11 (prone group) after cell injection. To this end, mice were anaesthetized with 2.5% isoflurane (Isoflurane CP, CP‐Pharma) in oxygen, shaved ventrally and dorsally and luciferin (D‐Luciferin potassium salt VivoTrace, Intrace Medical, Switzerland), and dissolved in PBS to reach a concentration of 30 mg mL^−1^, was injected s.c. in the neck area at a dose of 120 mg kg^−1^. Mice were measured in prone position; bioluminescent measurement was performed for 30 min in stage C to capture the signal peak of the luciferin kinetics 15–20 min after luciferin injection. For exposure, auto settings were chosen.

Mice were anaesthetized as mentioned above. Polymers were reconstituted in 0.9% NaCl and a dosage of 20 mg kg^−1^ was injected i.v. Fluorescence imaging was started 5 min after injection and data was acquired for 60 min. Automated exposure time was chosen. The following filter combinations were used: excitation (Ex; 30 nm bandwidth) at 605 nm with emissions (Em; 20 nm bandwidth) at 660, 680 and 700 nm and Ex at 640 nm with Em at 680, 700, 720 and 740 nm. Mice were first measured either in supine or prone position alongside a control animal. Afterward, mice were flipped over (from prone in supine or from supine in prone). An overdose of ketamine/xylazine was injected and an open body cavity fluorescence measurement was performed. Blood was drawn by cardiac puncture and left for clotting to obtain serum; urine and feces were gathered. Organs as well as tumor were collected, placed on a non‐reflective plate and measured. Finally, organs and tumors were embedded in Cryomolds using TissueTec OCT (Sakura Finetek Europe B.V., Umkirch, Germany), frozen on dry ice, and stored at −80 °C.

Multiple filter pairs were used for data acquisition. Therefore, spectral unmixing was performed for analysis of the fluorescence imaging data, as described in Geyer et al., 2017.^[^ ^61^ ^]^ In brief, the highest Cy5‐signal was marked on the treated animal, while the highest autofluorescence signal was selected on the control animal. Subsequently, the autofluorescence was automatically subtracted from the Cy5‐signal. ROIs were placed manually over the animals. For supine position, the ROIs thorax, upper abdomen and lower abdomen were selected, while for prone, thorax, kidney, tumor and tumor control were chosen (Figure S23, Supporting Information). Shown values are blotted as radiant efficiency [p/s/cm^2^/sr]/[µW/cm^2^]. Living Image version 4.5.2 was used for analysis.

### Histological Evaluations

The OCT blocks containing organs were sliced to 5 µm sections at −20 °C using a cryomicrotome (Cryostar NX70, ThermoScientific, USA). Afterward, the cryo slides were defrosted and the tissue was fixed with 4% PFA in PBS. Then, all tissues were stained with 1.5 µg mL^−1^ DAPI (diluted in PBS) for 15 min at r.t. and mounted in non‐hardening mounting medium (Vectashield Mounting Media, Vector, USA). Finally, the full tissue fluorescence images were taken using a fully automated 12 slide stage (TissueFAXS, TissueGnostics) on a Zeiss observer microscope, equipped with a fluorescence light source (X‐Cite Series 120 PC Q) and a high‐speed fluorescence camera (Hamamatsu Orca Flash 4.0), by a 20×/0.5 EC‐Plan‐Neofluor lens. Images were processed with the open source software TissueFAXS Viewer (TissueGnostics). In addition, H&E staining was performed as previously described in Feldman and Wolfe et al.,^[^ ^62^ ^]^ followed by scanning with a 3D Histech Microscopic High Throughput slide scanner for Brightfield. The Papanicolaou's solution 1a Harris’ hematoxylin solution was purchased from MERCK, the Scott's solution was bought from Morphisto and the Eosin Y disodium salt was purchased form MERCK.

### Statistics

All data are presented as mean ± SD. Comparison between groups was analyzed by mixed effects analysis or ordinary one‐way ANOVA with Dunnett's correction. Statistical analysis was performed using Prism 8 (GraphPad) (*p < 0.05, **p < 0.01, ***p < 0.001, and ****p < 0.0001)

## Conflict of Interest

The authors declare no conflict of interest.

## Supporting information

Supporting Information

## Data Availability

The data that support the findings of this study are available in the supplementary material of this article.
