# Supplementary material for: Degradable Bottlebrush Polypeptides and the Impact of their Architecture on Cell Uptake, Pharmacokinetics, and Biodistribution In Vivo
Source: Small. 2023 Feb 26;19(22):2300767. doi: 10.1002/smll.202300767 (PMC11475343; doi:10.1002/smll.202300767)
Supplement: Supplementary file 1 — Supporting Information [file SMLL-19-2300767-s001.pdf]

NANO MICRO small

Supporting Information

for *Small,* DOI: 10.1002/smll.202300767

Degradable Bottlebrush Polypeptides and the Impact of their Architecture on Cell Uptake, Pharmacokinetics, and Biodistribution In Vivo

Paul Strasser, Bianca Montsch, Silvia Weiss, Haider Sami, Christoph Kugler, Sonja Hager, Hemma Schueffl, Robert Mader, Oliver Brüggemann, Christian R. Kowol, Manfred Ogris, Petra Heffeter,* and Ian Teasdale**

Electronic Supplementary Information

Degradable bottlebrush polypeptides and the impact of their architecture on cell uptake, pharmacokinetics and biodistribution *in vivo*

Paul Strasser, Bianca Montsch, Silvia Weiss, Haider Sami, Christoph Kugler, Sonja Hager, Hemma Schueffl, Robert Mader, Oliver Brüggemann, Christian R. Kowol, Manfred Ogris, Petra Heffeter and Ian Teasdale

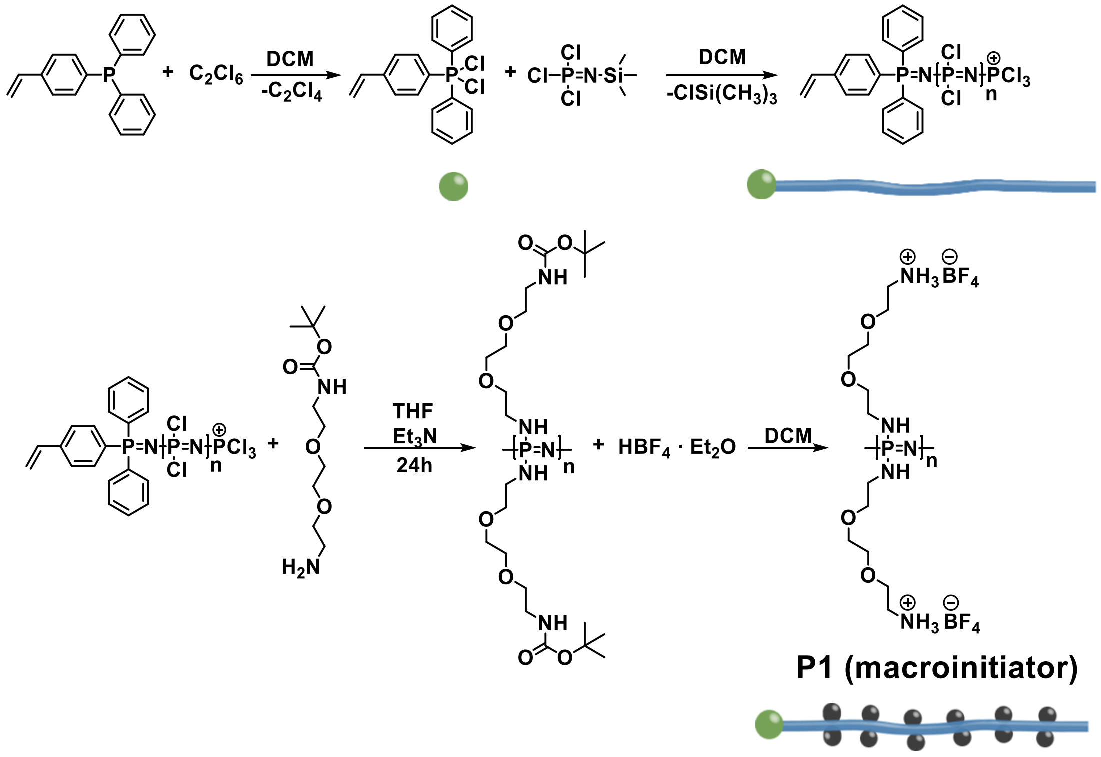

Figure S1. Schematic synthesis pathway for the PPz macroinitiator **P1**.

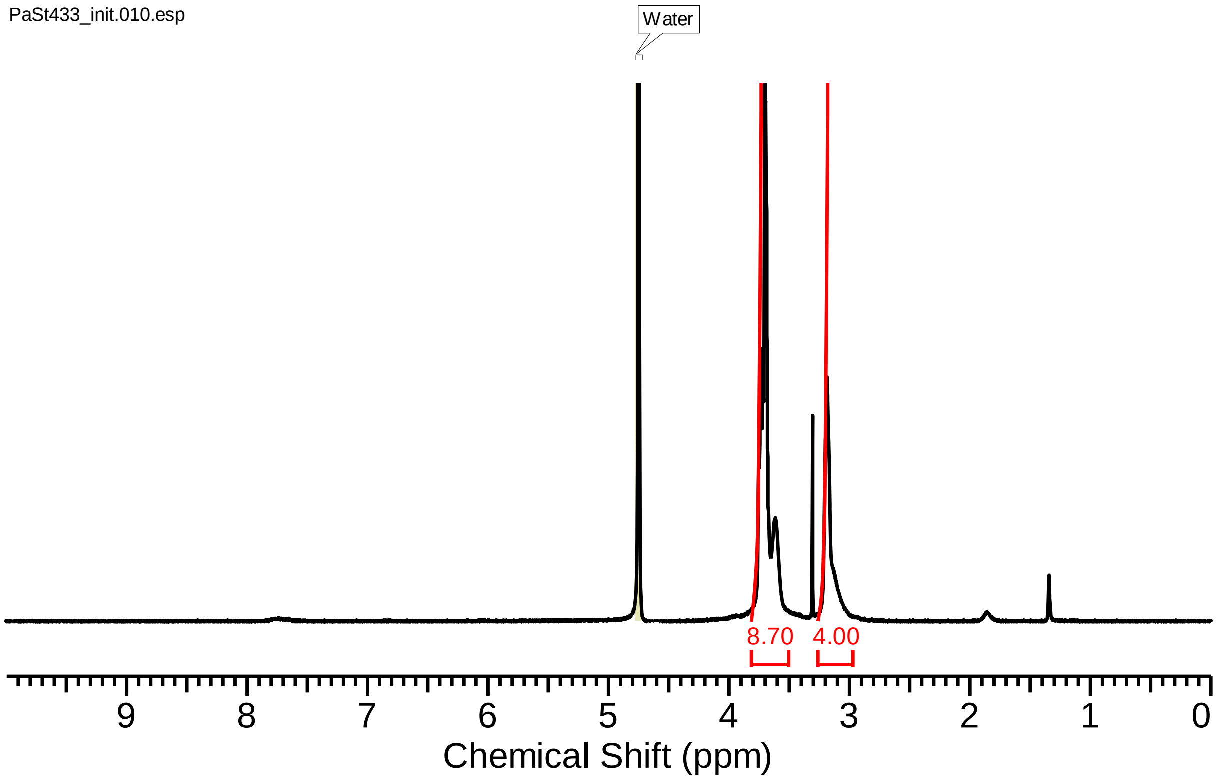

Figure S2. ^{1}H-NMR spectrum of macroinitiator **P1** in D_2O.

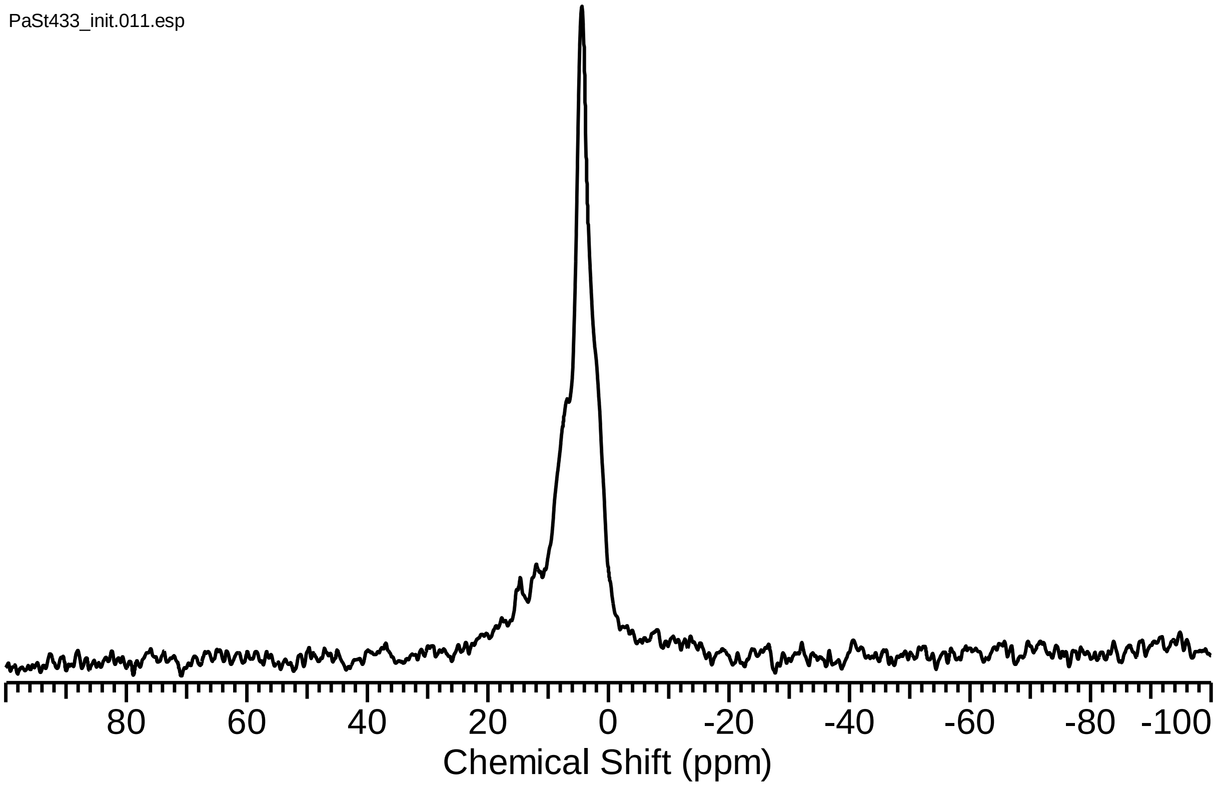

Figure S3. ^{31}P-NMR spectrum of **P1** in D_2O.

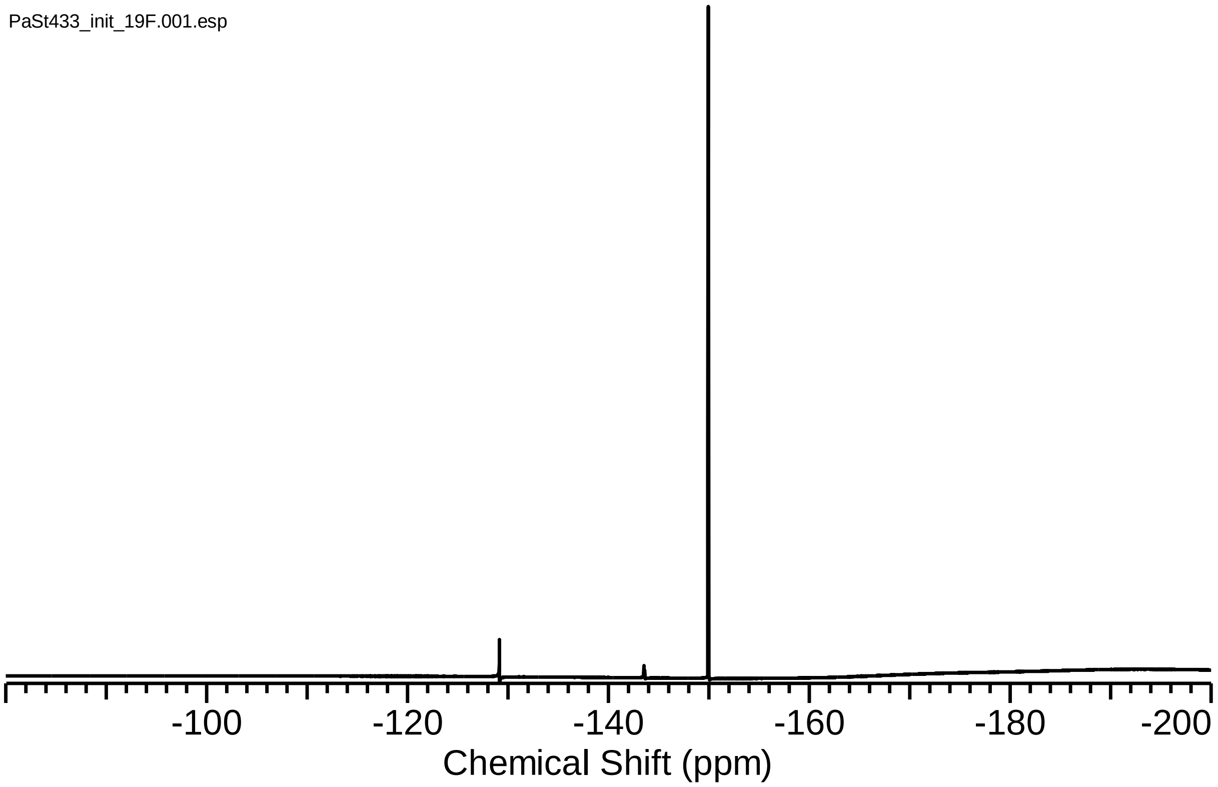

Figure S4. ^{19}F-NMR spectrum of **P1** in D_2O.

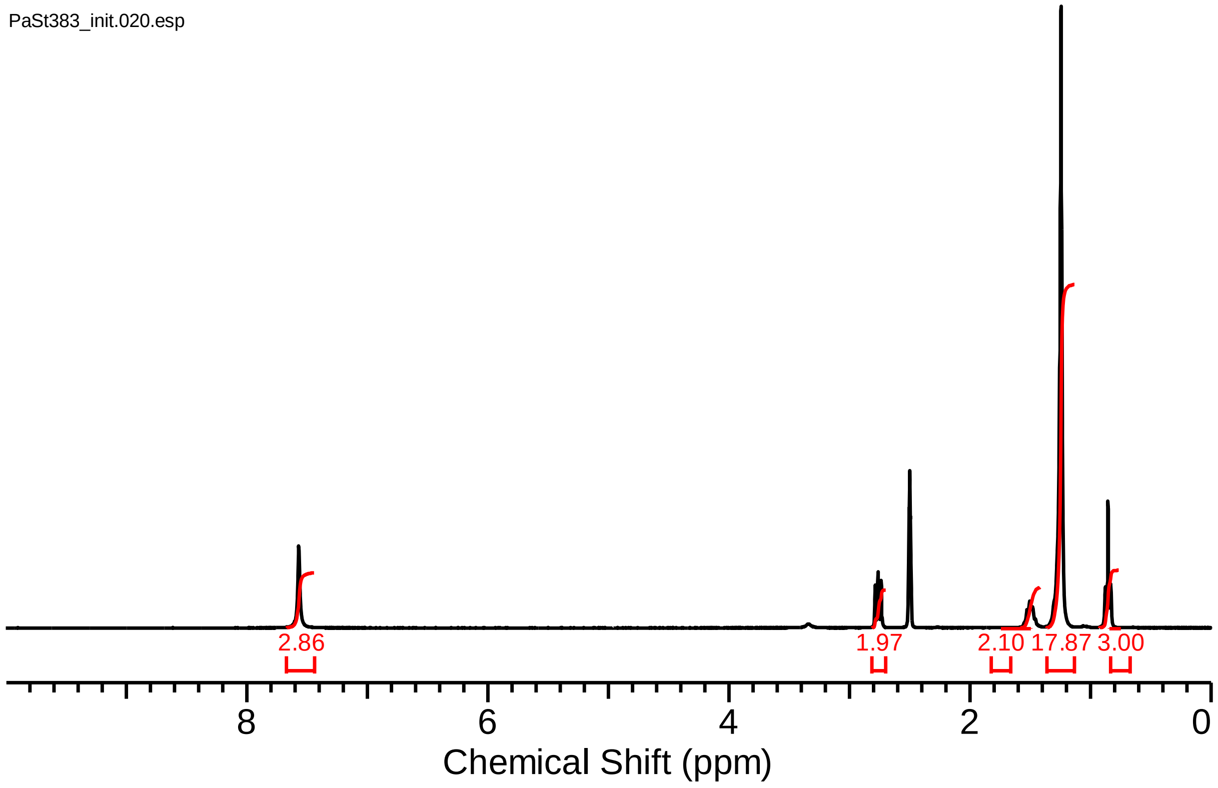

Figure S5. ^{1}H-NMR spectrum of the dodecylammonium BF_4 salt (initiator) in DMSO-d_6.

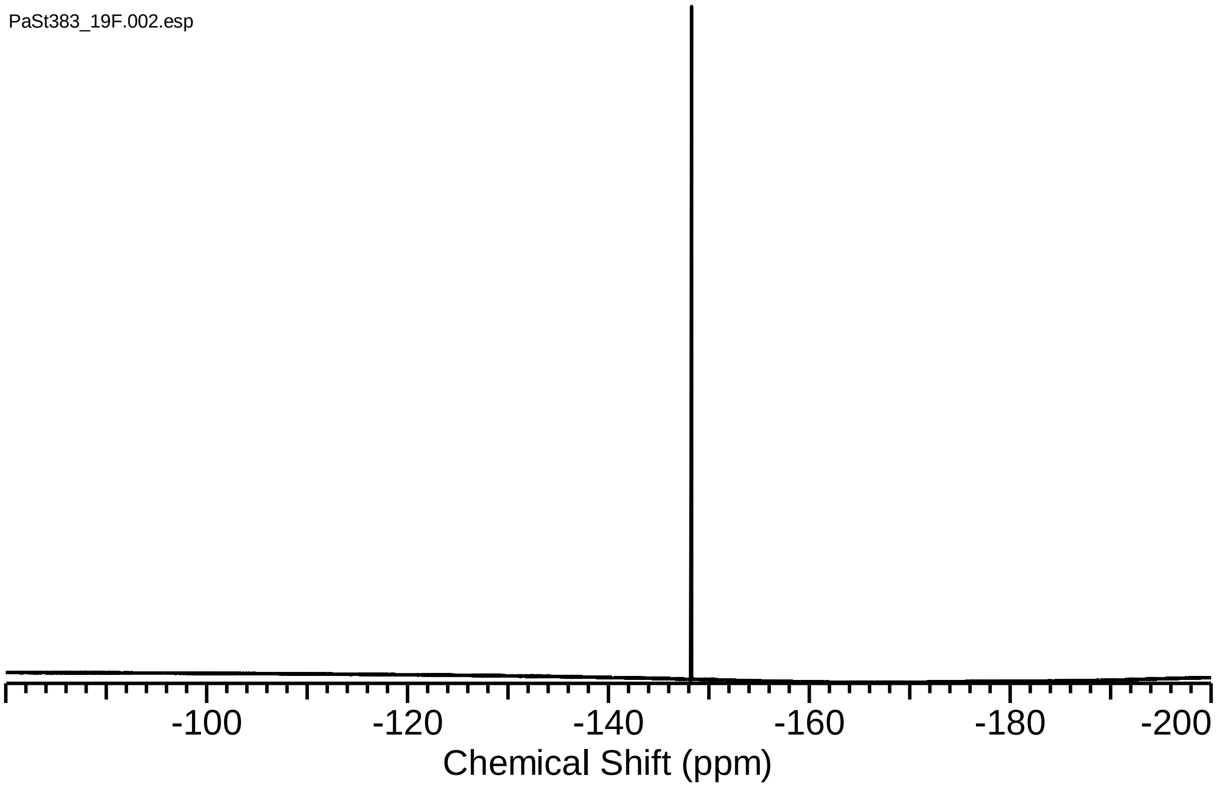

Figure S6. ^{19}F-NMR spectrum of the dodecylammonium BF_4 salt (initiator) in DMSO-d_6.

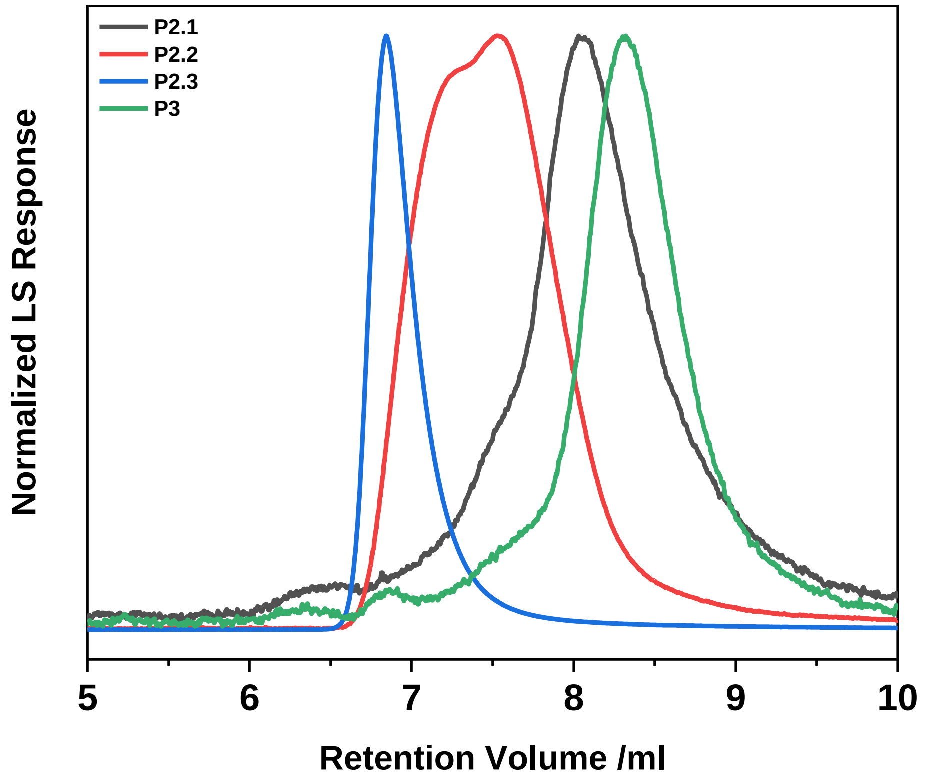

Figure S7. SEC LS-traces of the benzyl-ester protected polymers at 2 mg/ml in DMF (10mM LiBr) without purification.

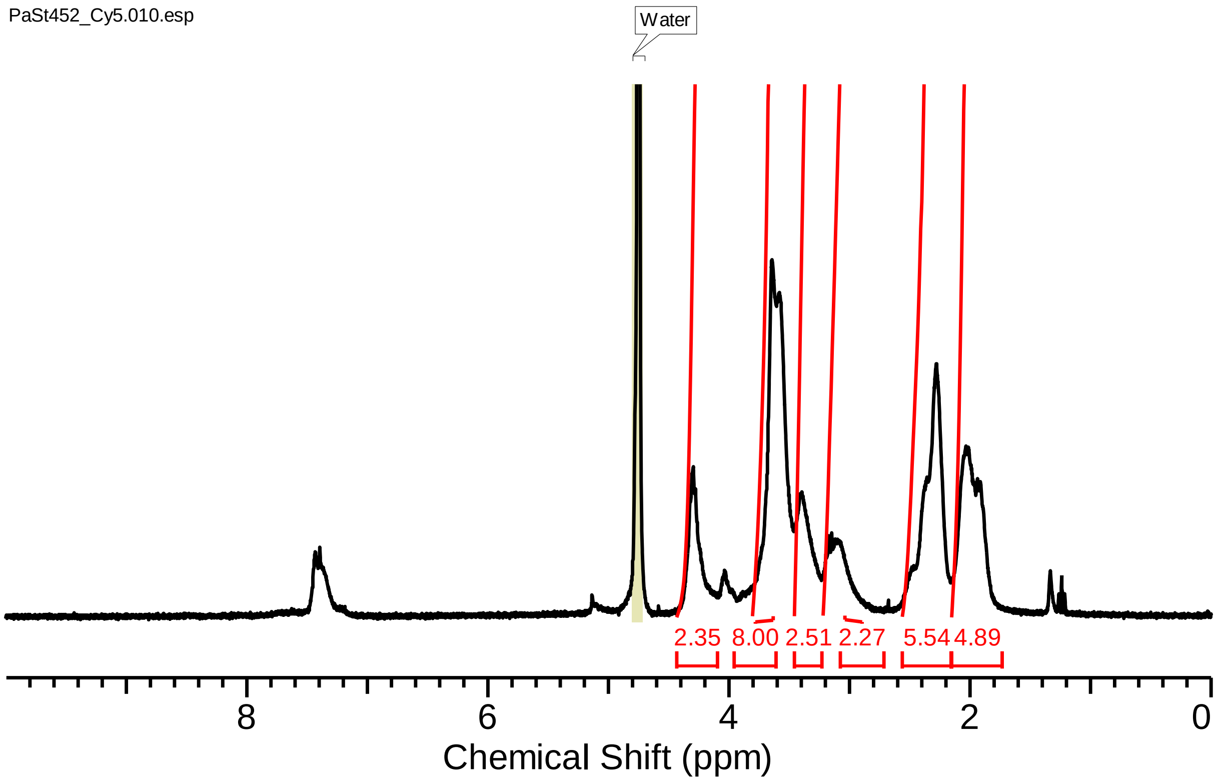

Figure S8. ^{1}H-NMR spectrum of the PPz-*g*-PGA bottlebrush polymer **P4.1** in D_2O.

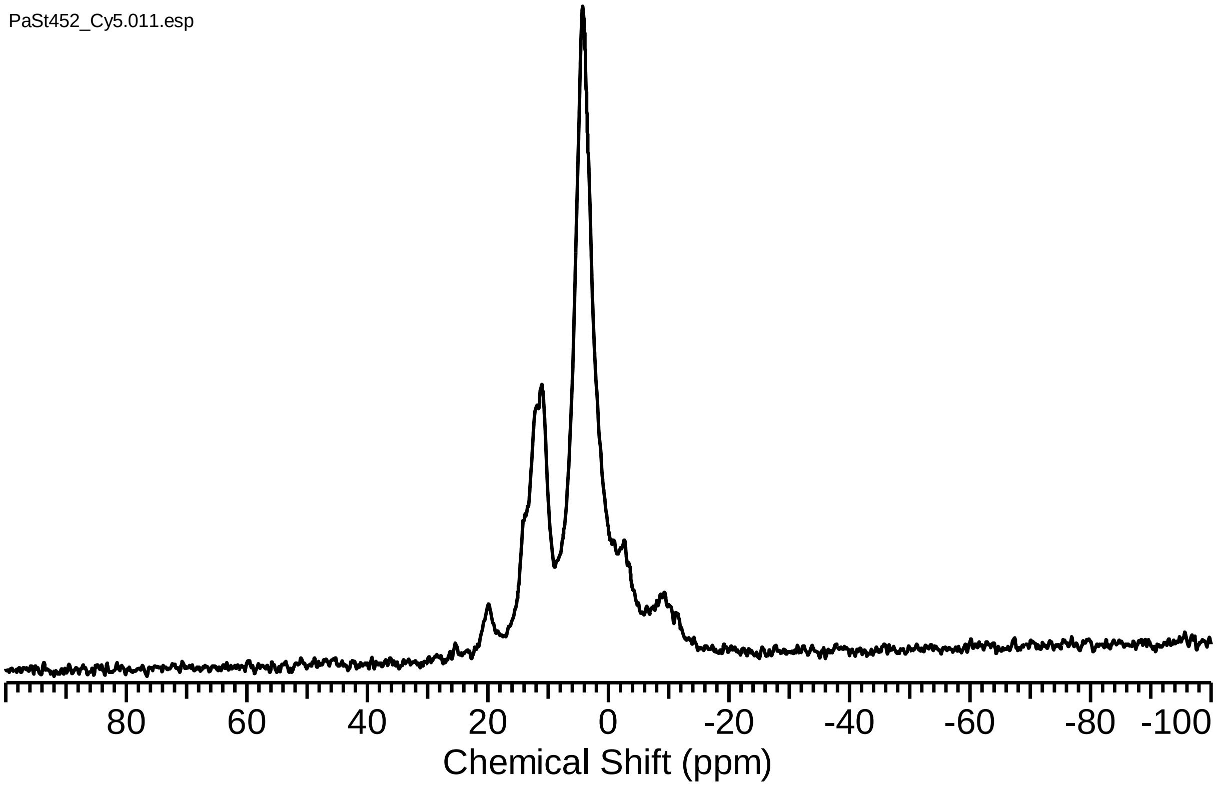

Figure S9. ^{31}P-NMR spectrum of the PPz-*g*-PGA bottlebrush polymer **P4.1** in D_2O.

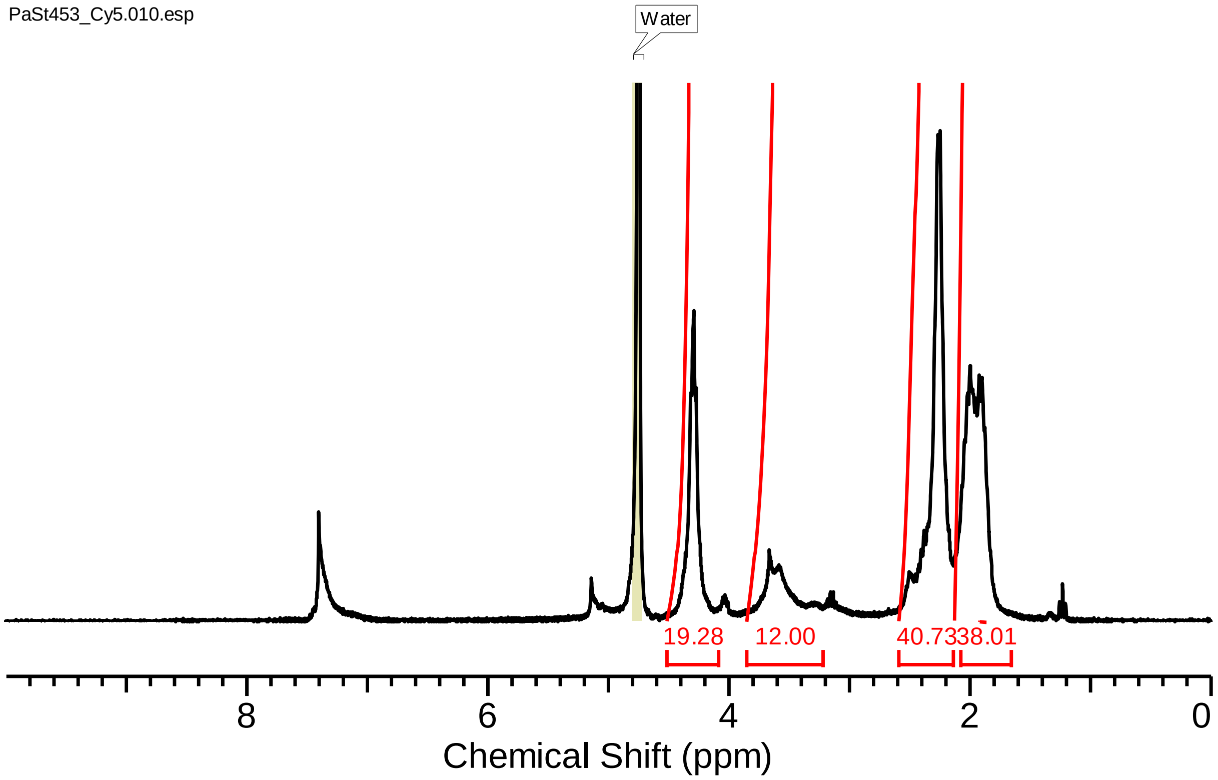

Figure S10. ^{1}H-NMR spectrum of the PPz-*g*-PGA bottlebrush polymer **P4.2** in D_2O.

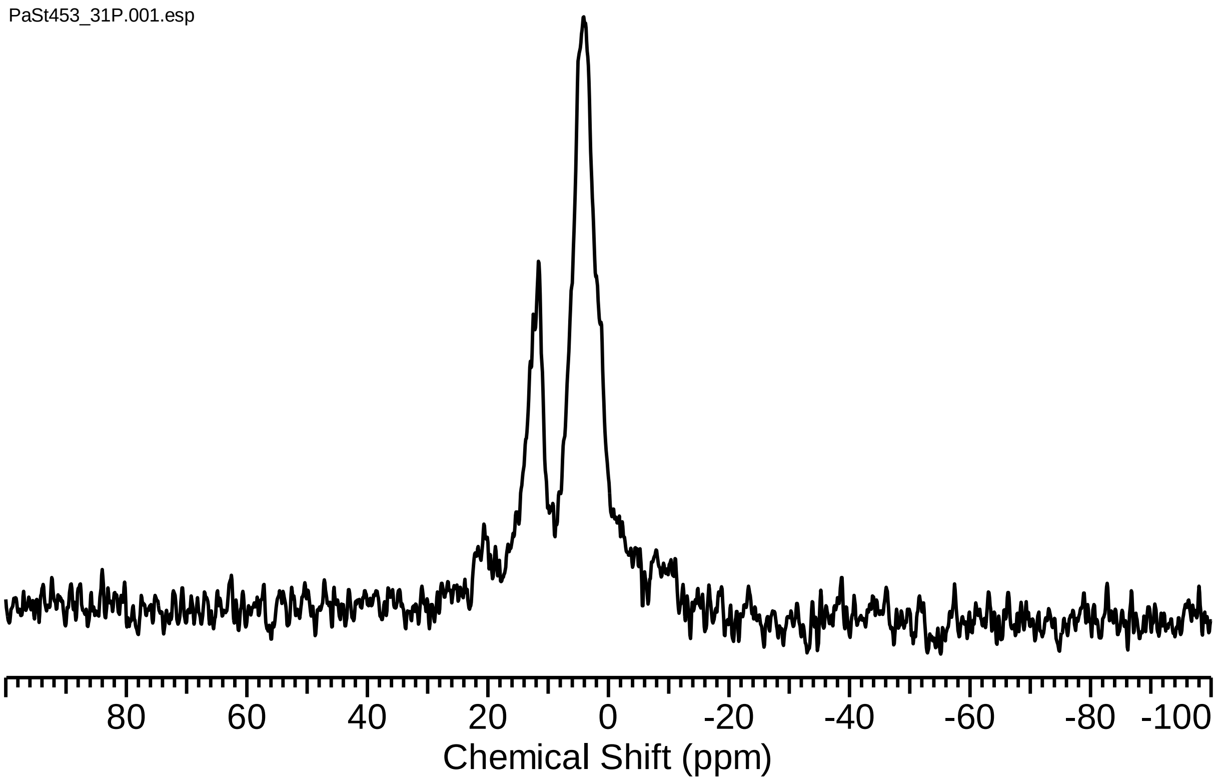

Figure S11. ^{31}P-NMR spectrum of the PPz-*g*-PGA bottlebrush polymer **P4.2** in D_2O.

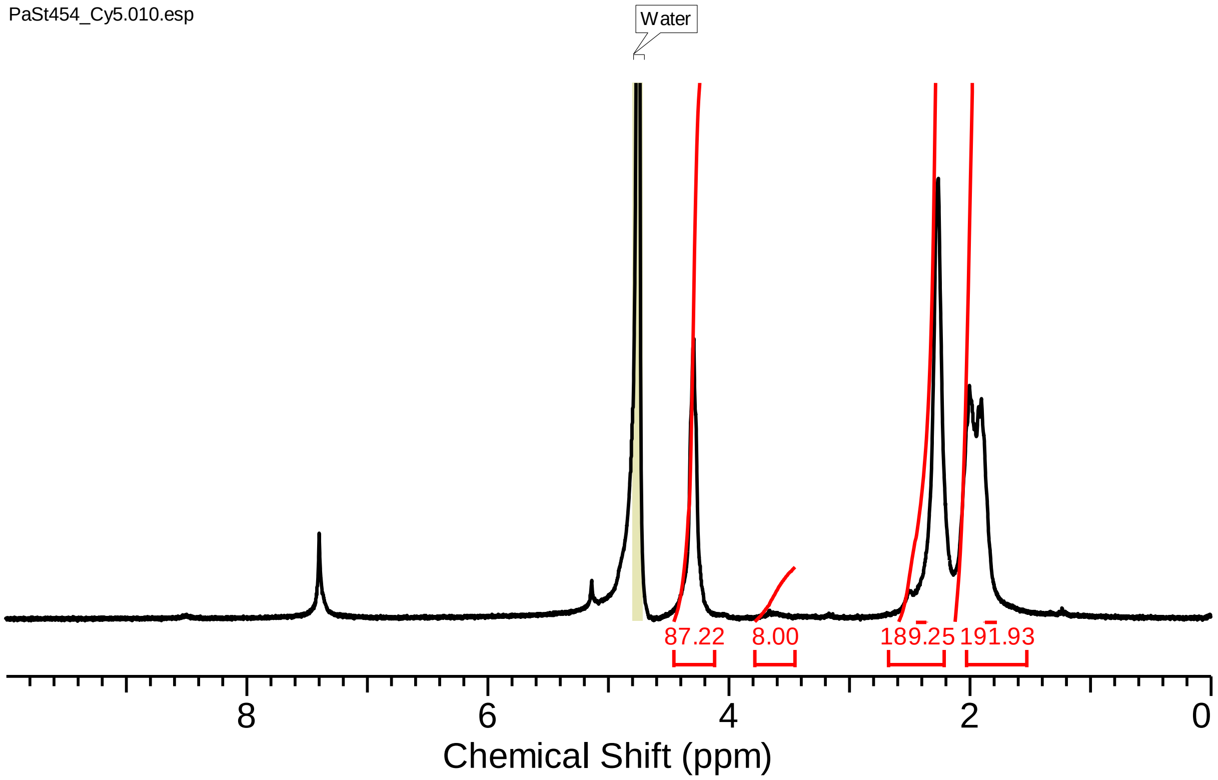

Figure S12. ^{1}H-NMR spectrum of PPz-*g*-PGA bottlebrush polymer **P4.3** in D_2O.

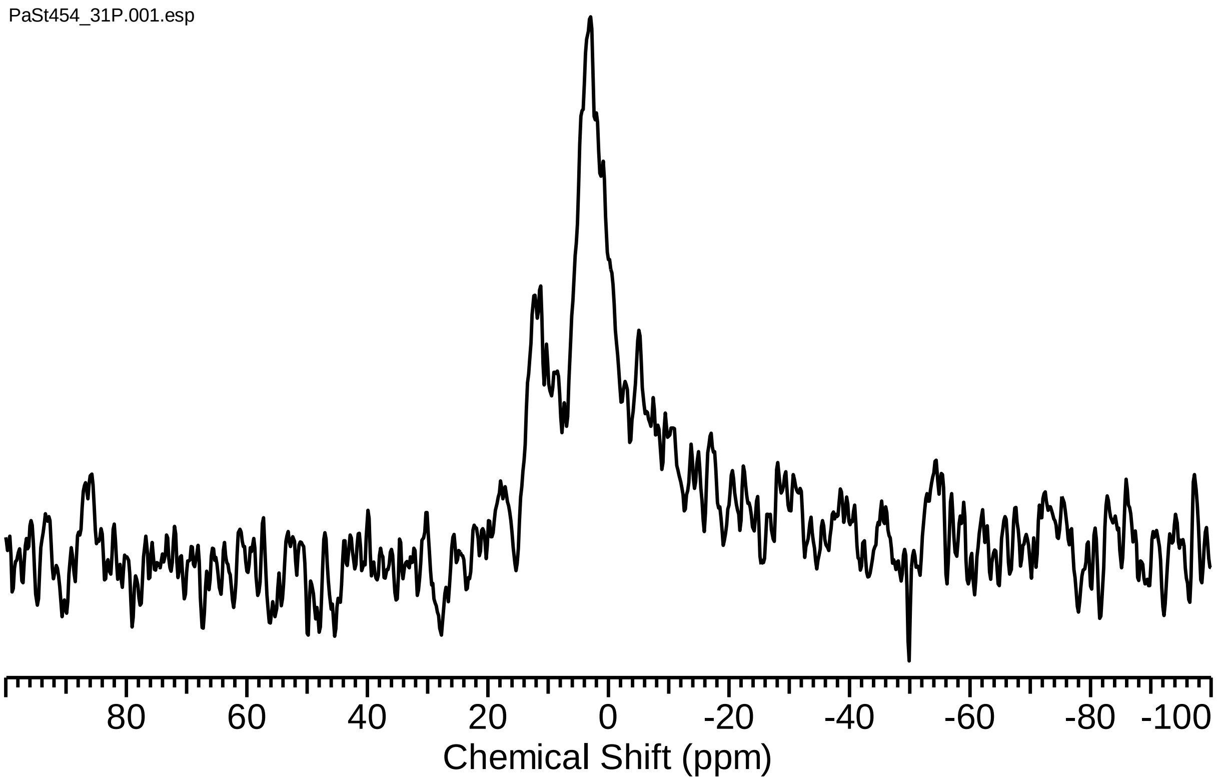

Figure S13. ^{31}P-NMR spectrum of the PPz-*g*-PGA bottlebrush polymer **P4.3** in D_2O.

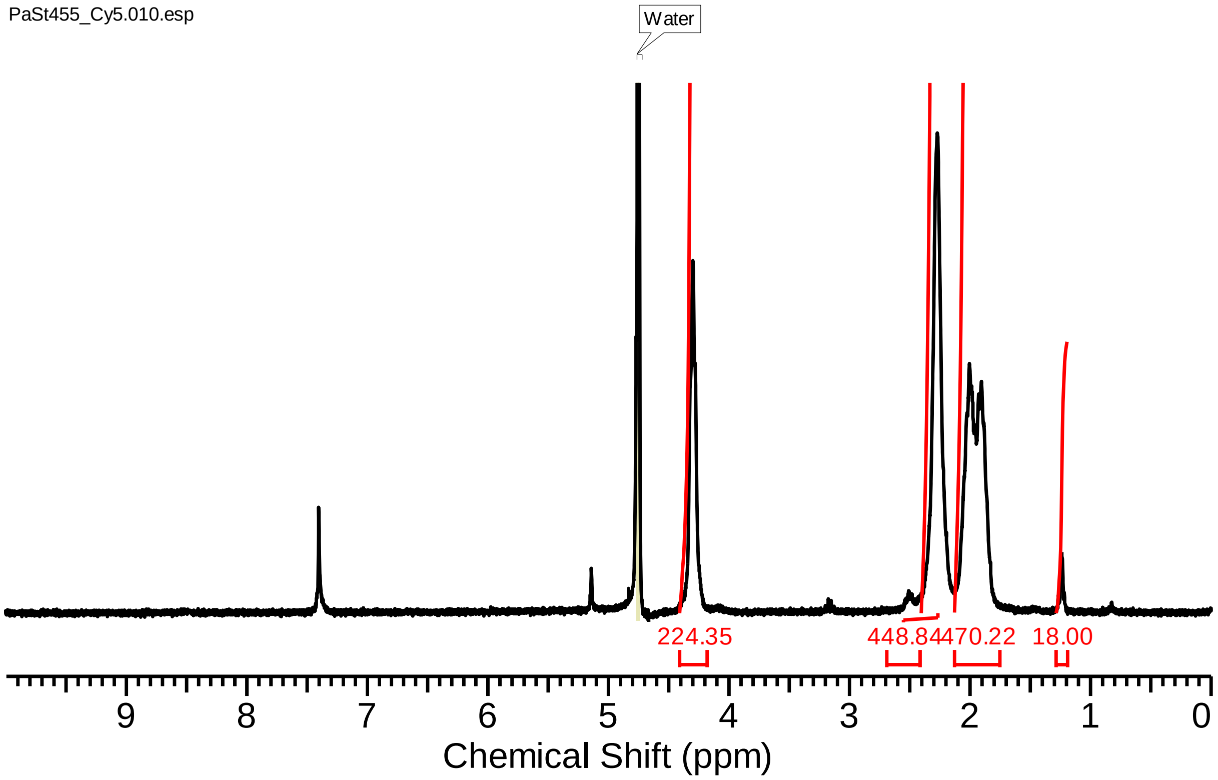

Figure S14. ^{1}H-NMR spectrum of the PGA bottlebrush polymer **P5** in D_2O.

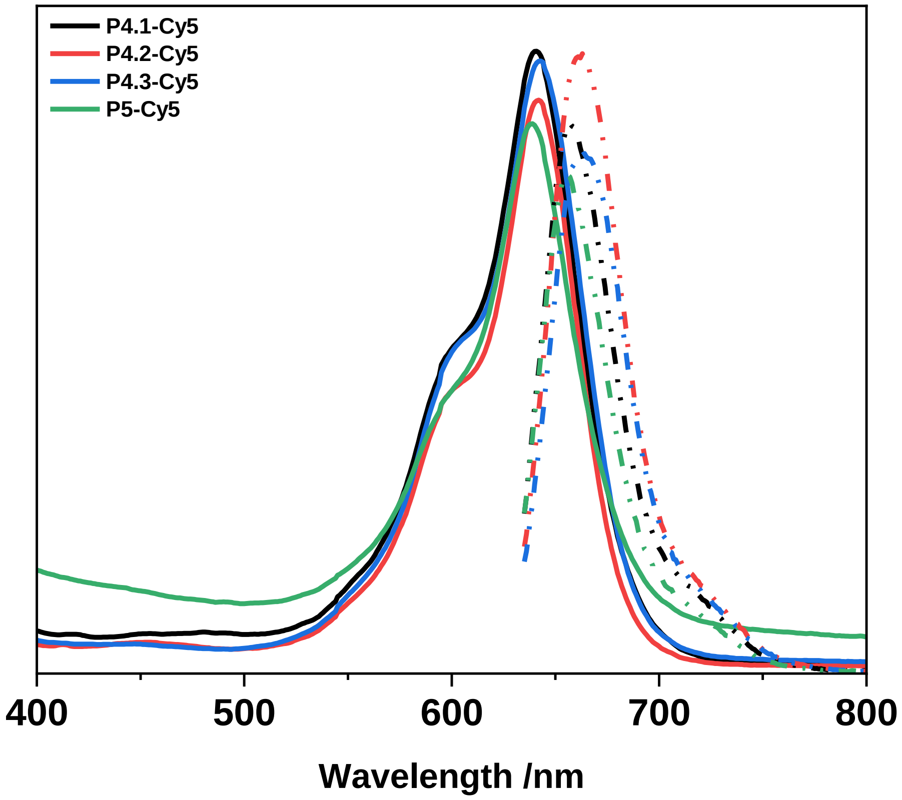

Figure S15. UV-Vis spectra of Cy5 labelled PPz-*g*-PGA bottlebrush (**P4.1**, **P4.2** and **P4.3**) and linear PGA (**P5**) polymers. Solid line: absorbance spectra; dotted line: emission spectra, excited at 625 nm.

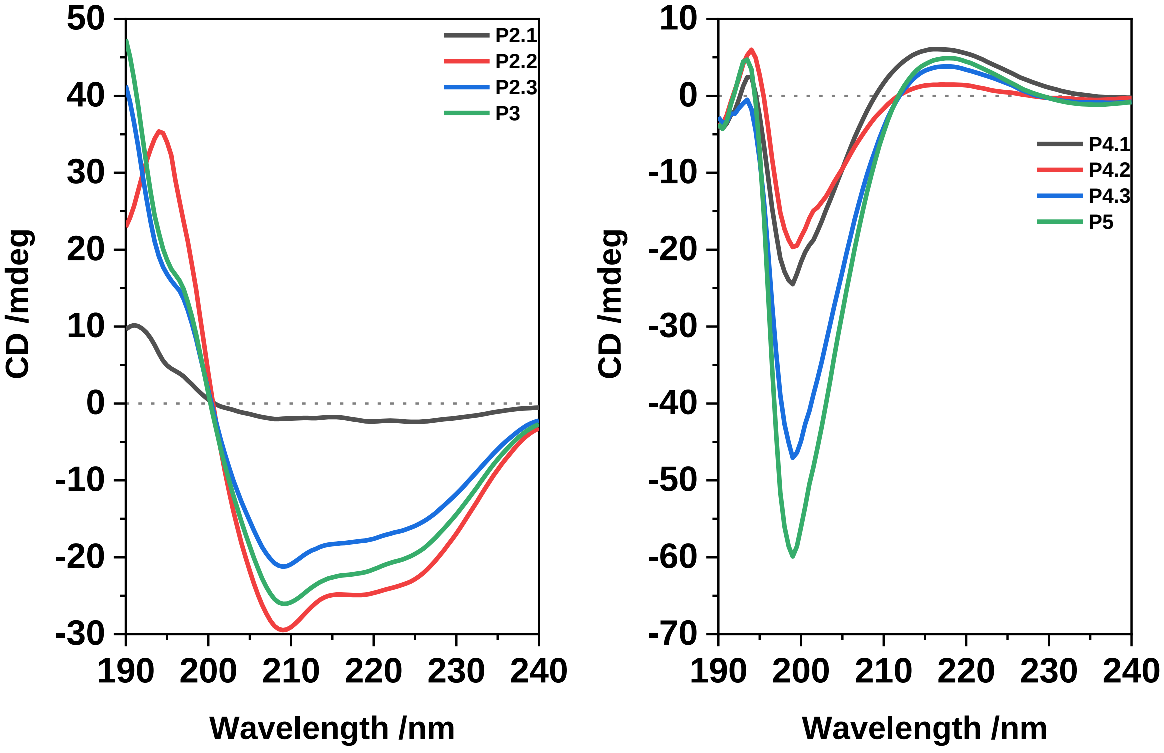

Figure S16. CD-spectra of bottlebrush polymers and linear PGA before (**P2.1**, **P2.2**, **P2.3** and **P3**), left, and after (**P4.1**, **P4.2**, **P4.3** and **P5**), right, deprotection of the side-chains. In 1,1,1-3,3,3-hexafluoroisopropanol at 20°C and PBS pH 7.4 at 37°C, respectively.

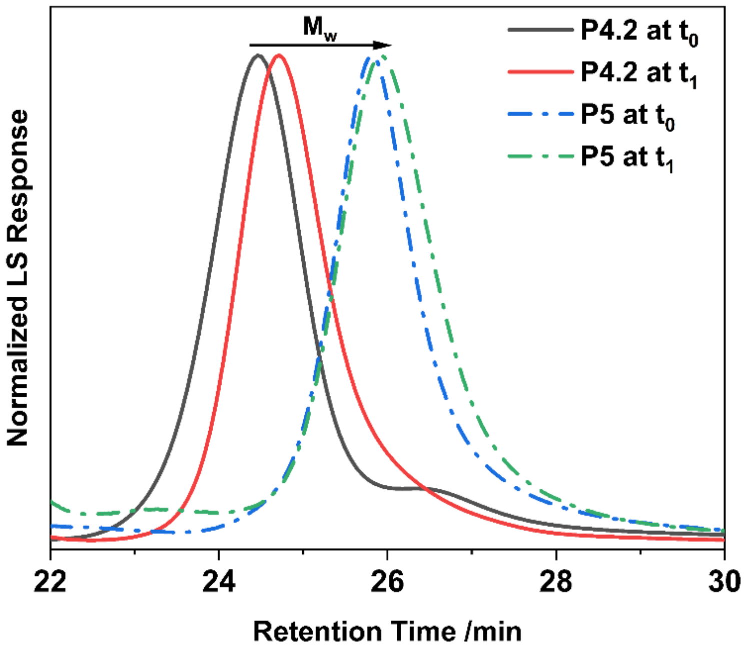

Figure S17. SEC-LS traces of the degradation of the PPz-*g*-PGA bottlebrush polymer **P4.2** and the linear analogue **P5** incubated in acetate buffer (pH 5.5) containing 10µg cathepsin B. $t_0 = 0$ d, $t_1 = 60$ d.

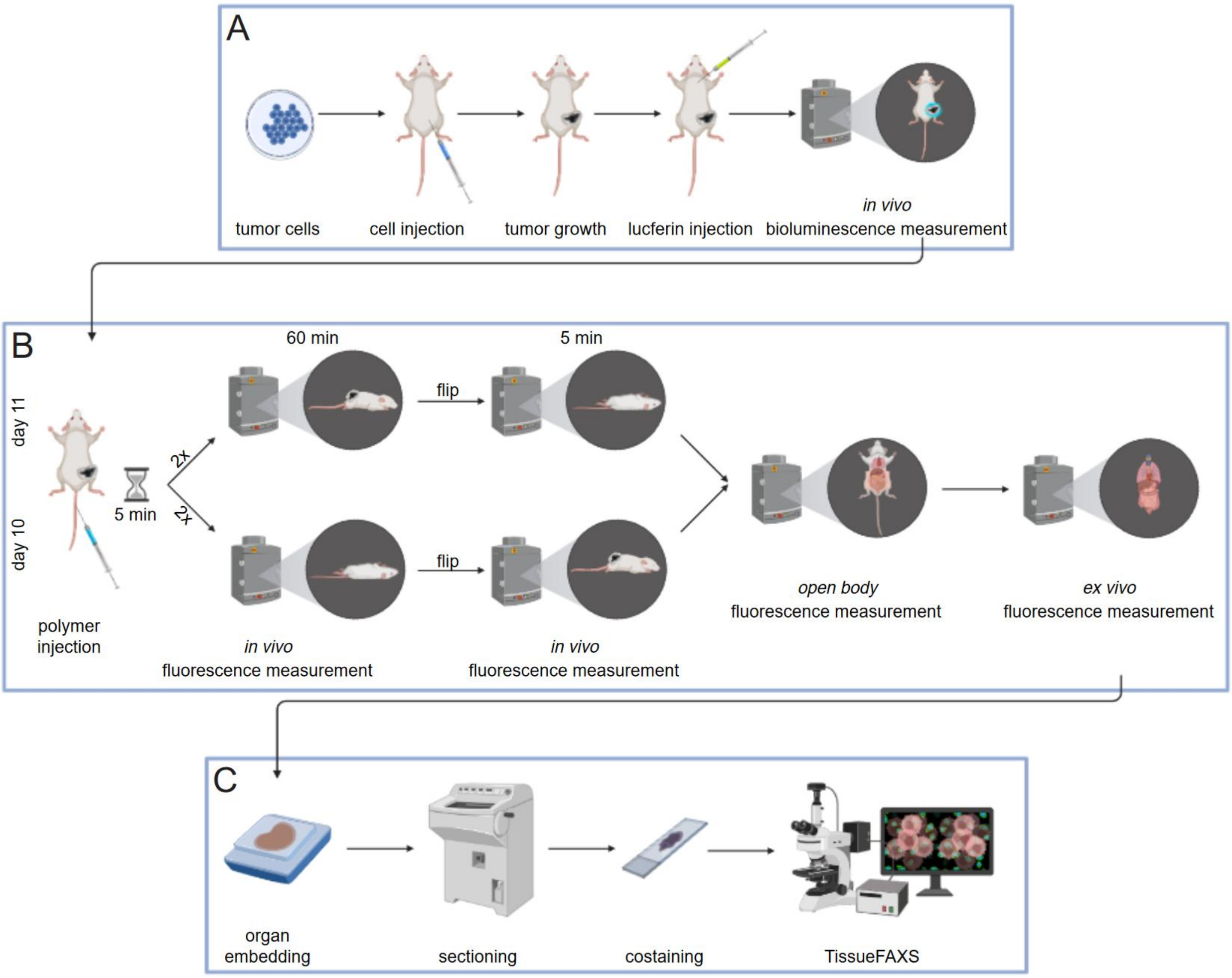

Figure S18. Overall workflow of biodistribution studies of Cy5-labeled bottlebrush polymers in $CT26_{luc}$ tumor bearing mice: (a) 5 x 10^5 luciferase-transfected $CT26_{luc}$ were s.c. injected into Balb/c mice on low fluorescent diet; tumor growth was regularly verified using bioluminescence imaging. (b) Cy5-labeled bottlebrush polymers **P4.1, P4.2, P4.3** or **P5** (100 mg/kg, dissolved in 0.9 % NaCl) were i.v. injected (n=4), fluorescence imaging was performed for 60 min either in prone (n=2) or supine (n=2) position. Animals were flipped (from prone to supine; from supine to prone) and measured for another 5 min. Open body imaging was performed, organs were collected and embedded after *ex vivo* fluorescence measurements. (c) Organs were sectioned using a cryostat, co-stained employing WGA and DAPI, and TissueFAXS measurements were performed. Created with BioRender.com.

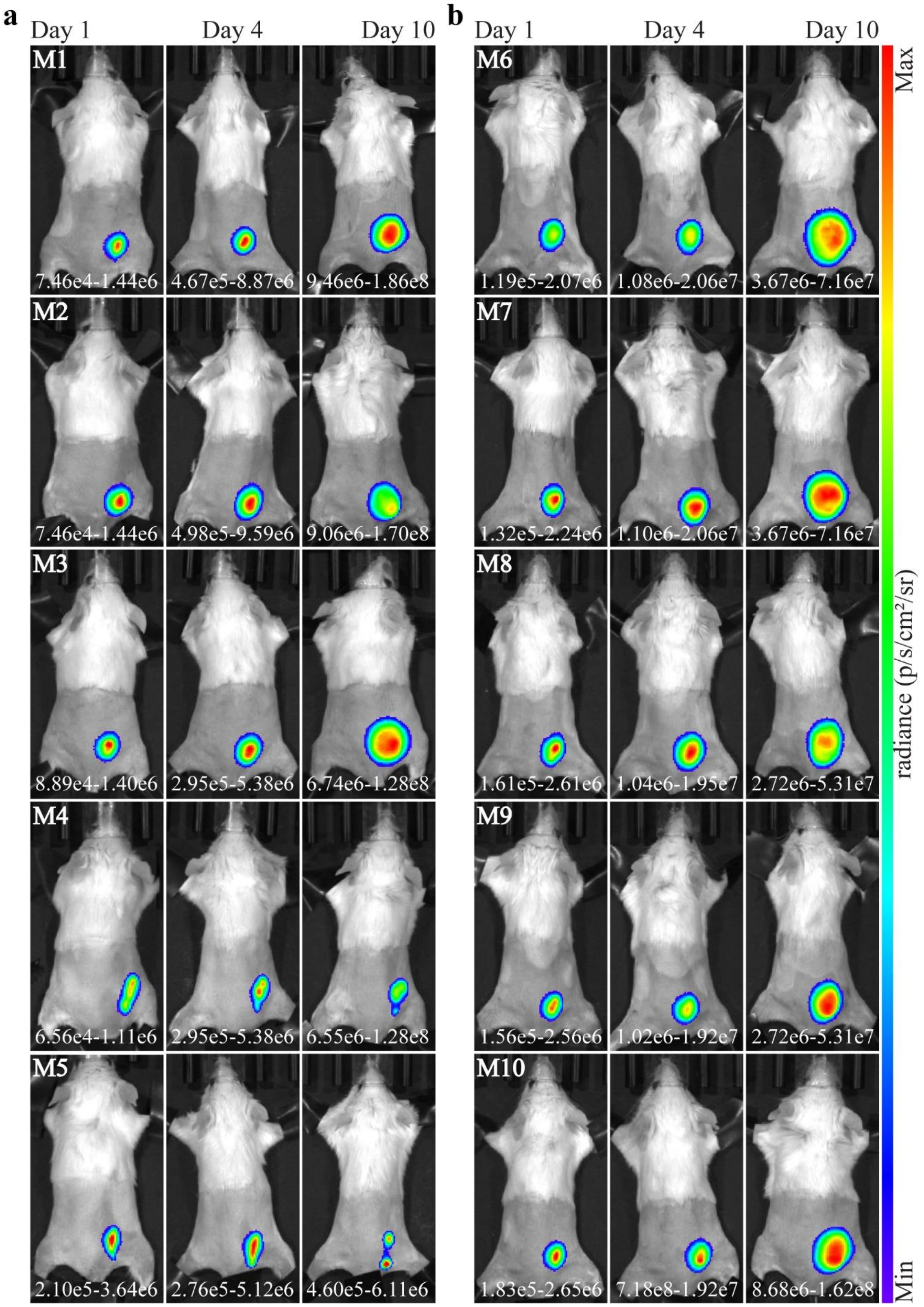

Figure S19 Bioluminescence imaging (BLI) based tumor visualization on day 1, 4 and 10 after implantation of $CT26_{luc}$ cells in Balb/c mice. Panel a and b show different sets of mice, Mx = mouse number x with minimum and maximum value for BLI signal in radiance mentioned on each image.

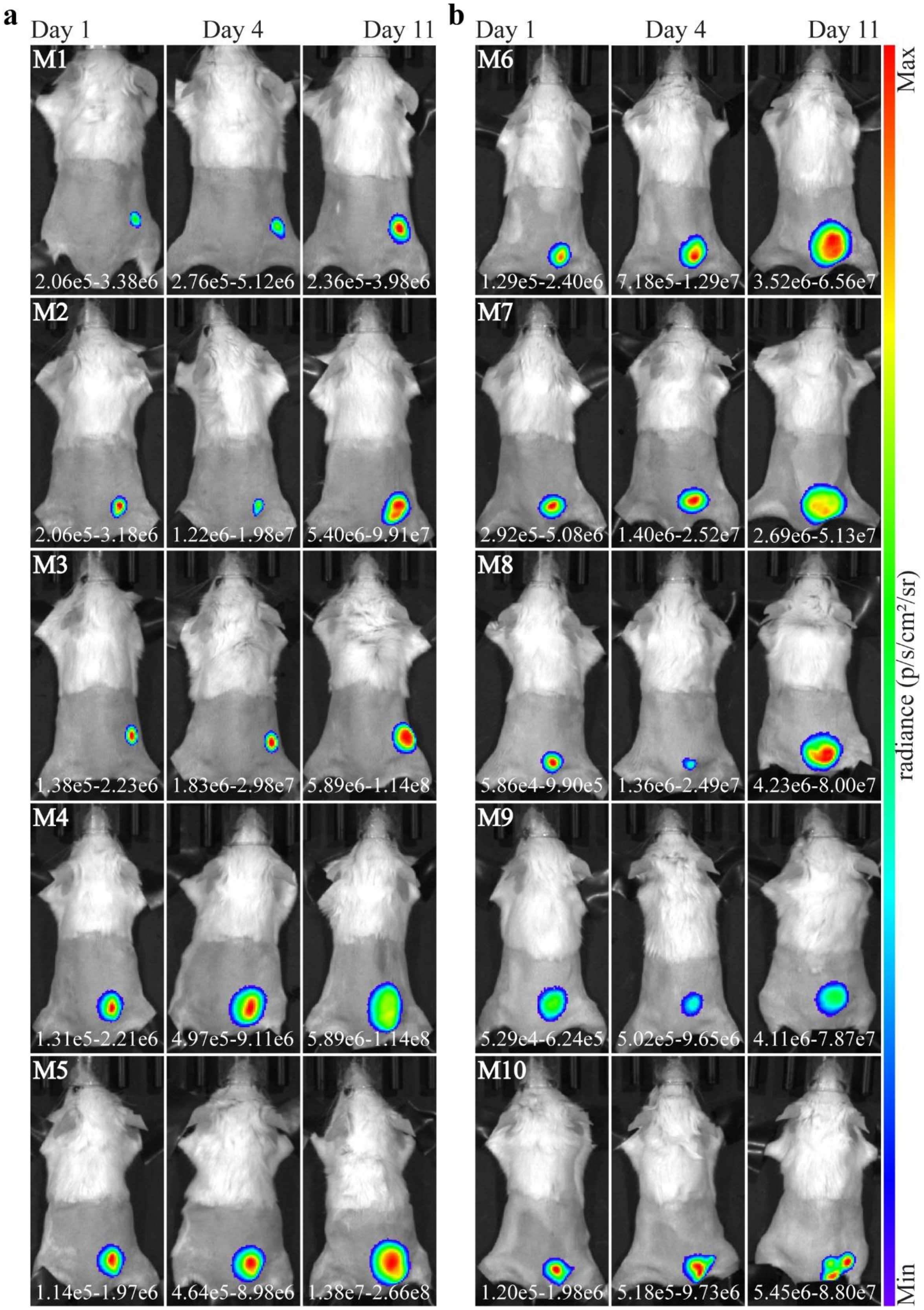

Figure S20. Bioluminescence imaging (BLI) based tumor visualization on day 1, 4 and 11 after implantation of $CT26_{luc}$ cells in Balb/c mice. Panel a and b show different sets of mice, Mx = mouse number x with minimum and maximum value for BLI signal in radiance mentioned on each image.

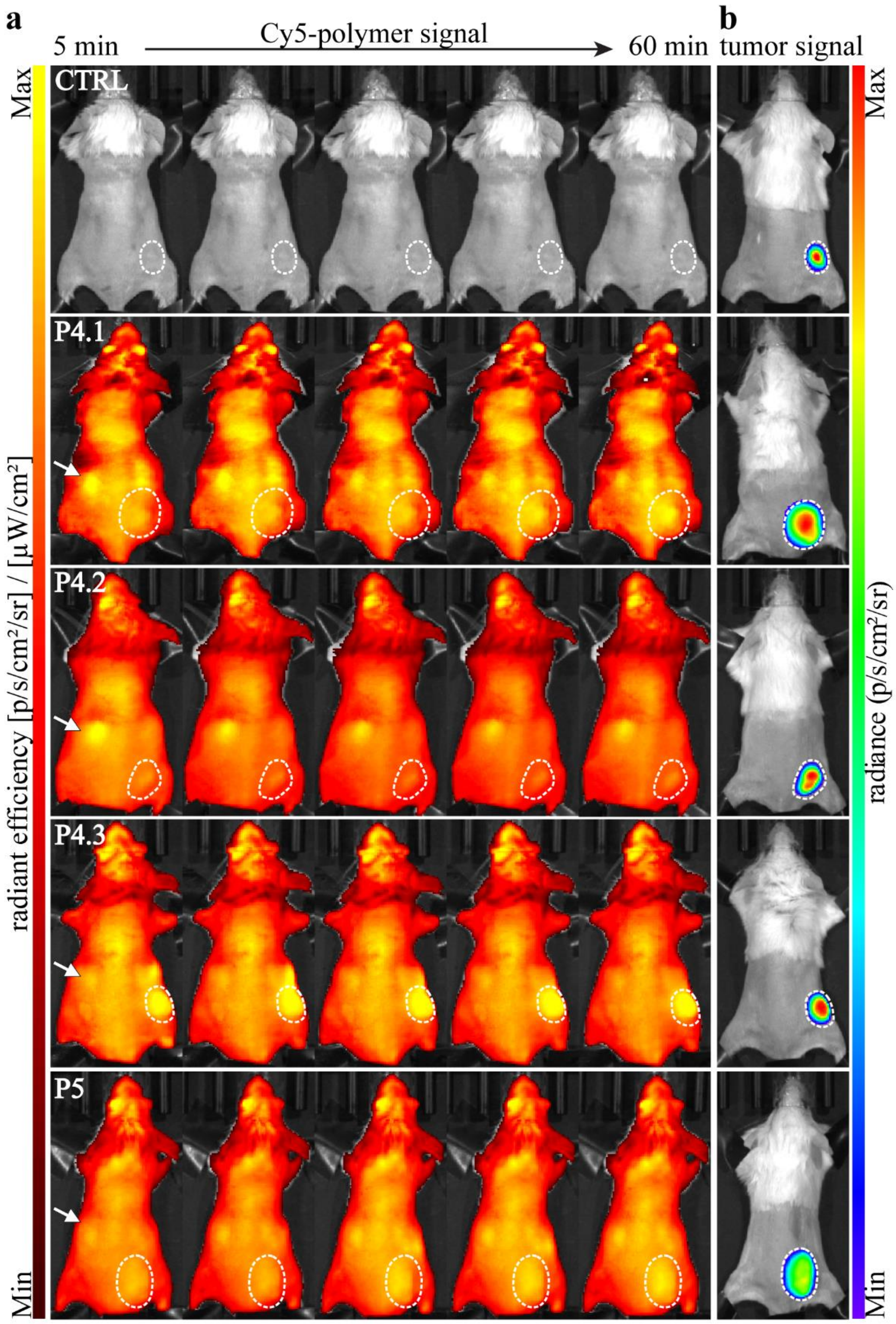

Figure S18. Biodistribution studies of different bottlebrush polymers in tumor-bearing mice. (a) **P4.1, P4.2, P4.3** or **P5** (100 mg/kg, Cy5-labeled, dissolved in 0.9 % NaCl) polymer was intravenously injected into $CT26_{luc}$ tumor bearing mice. Fluorescence imaging was performed for 60 min in prone position and results of 5, 15, 30, 45 and 60 min after polymer injection are shown. (b) Bioluminescence imaging was done just before Cy5-polymer administration for visualizing the tumor location of the same animal. Arrows indicate the position of the kidneys; tumors are encircled in white.

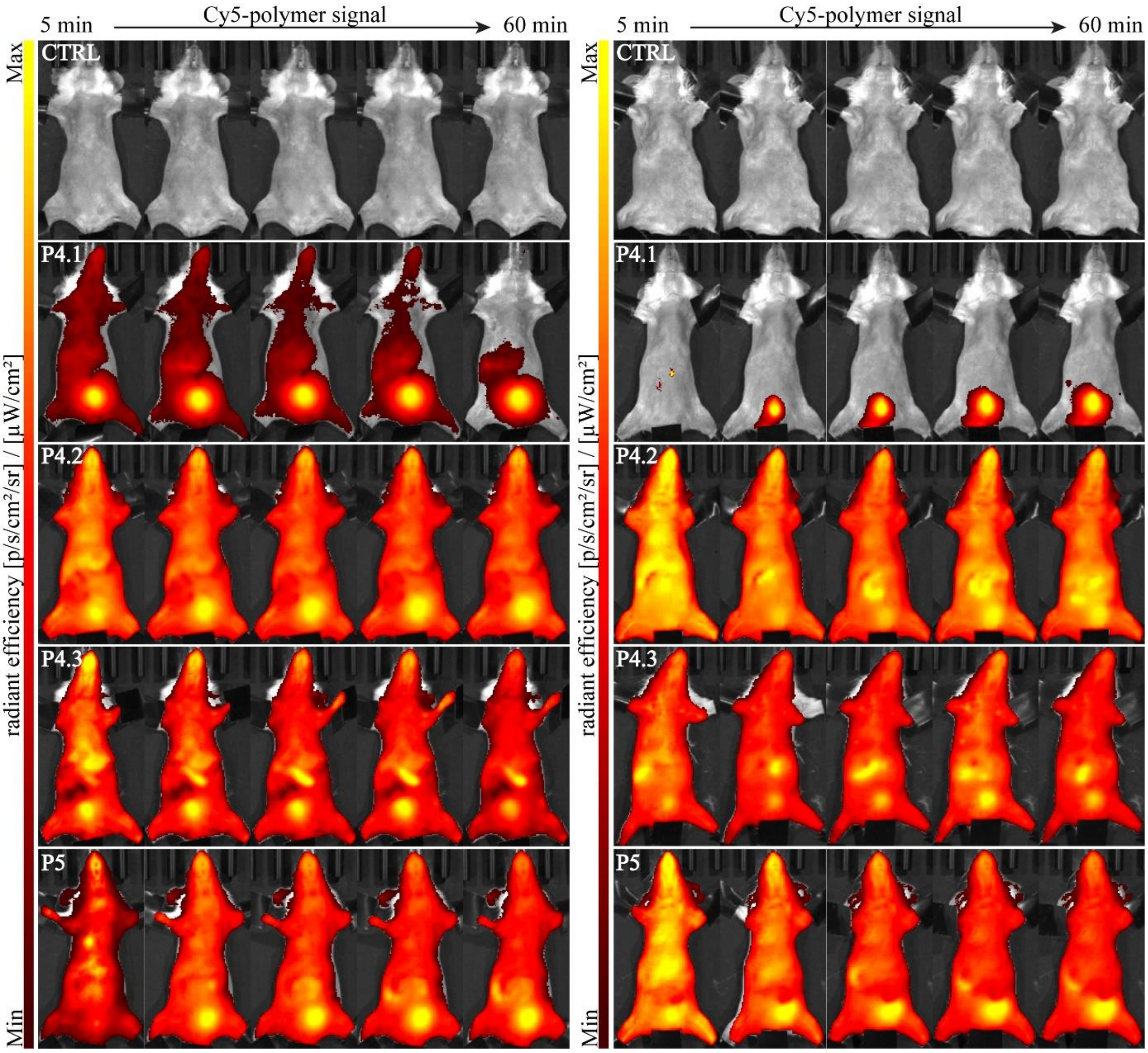

Figure S19. Biodistribution studies of different bottlebrush polymers in two tumor-bearing mice. **P4.1, P4.2, P4.3** or **P5** (100 mg/kg, Cy5-labeled, dissolved in 0.9 % NaCl) polymer was intravenously injected into CT26$_{luc}$ tumor bearing mice. Fluorescence imaging was performed for 60 min in supine position and results of 5, 15, 30, 45 and 60 min after polymer injection are shown.

a

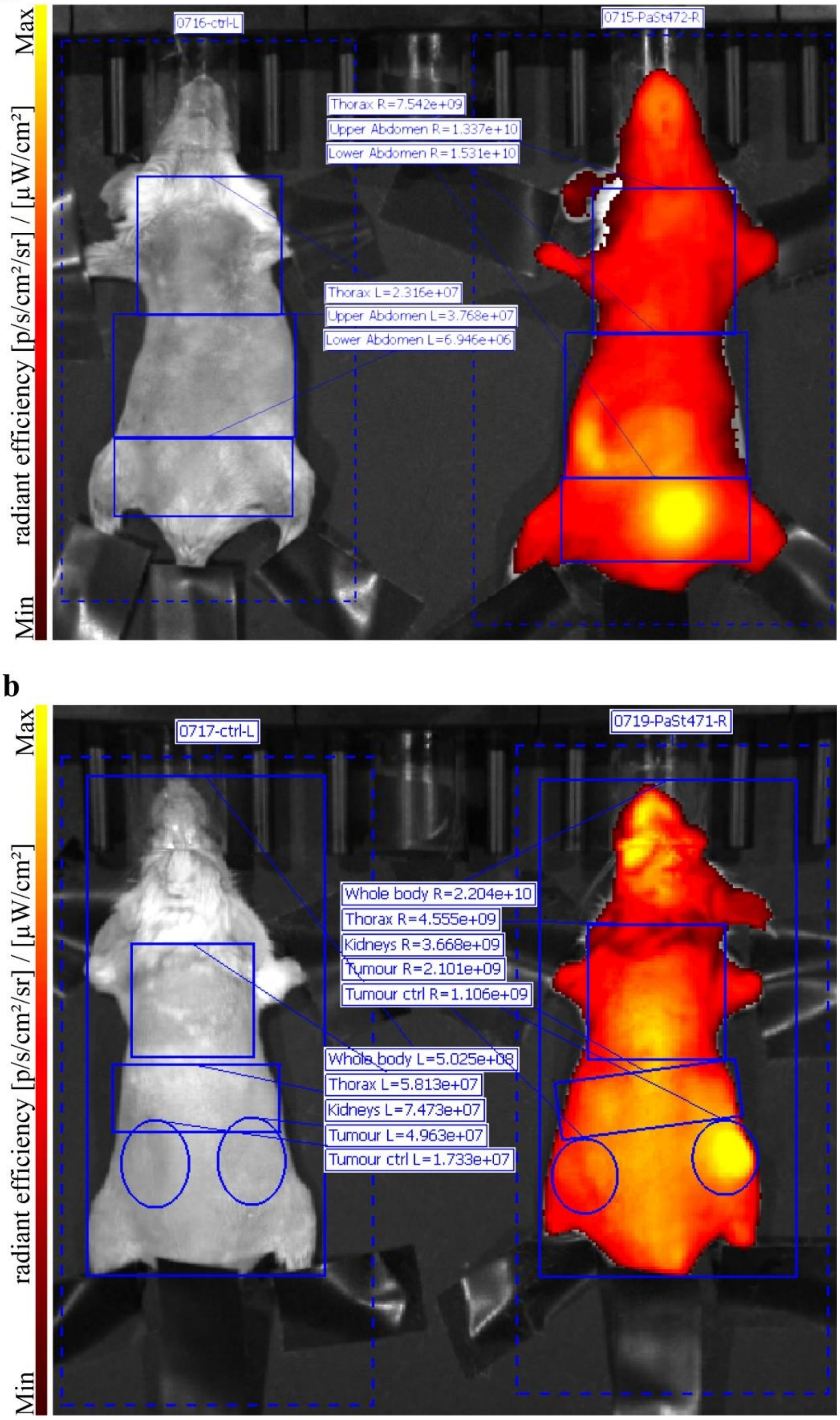

Figure S23. Representative example of region of interest (ROI) placement for fluorescence imaging analysis: in supine position (a), the ROIs “Thorax”, “Upper Abdomen” and “Lower Abdomen” were placed, while in prone position (b) the regions of interest were “Whole body”, “Thorax”. “Kidneys”, “Tumor” and “Tumor ctrl”. The ROI “Tumor ctrl” has the same size as “Tumor” and is placed on the same mouse but an area without tumor. The ROI placement for treated and untreated mice was identical.

a

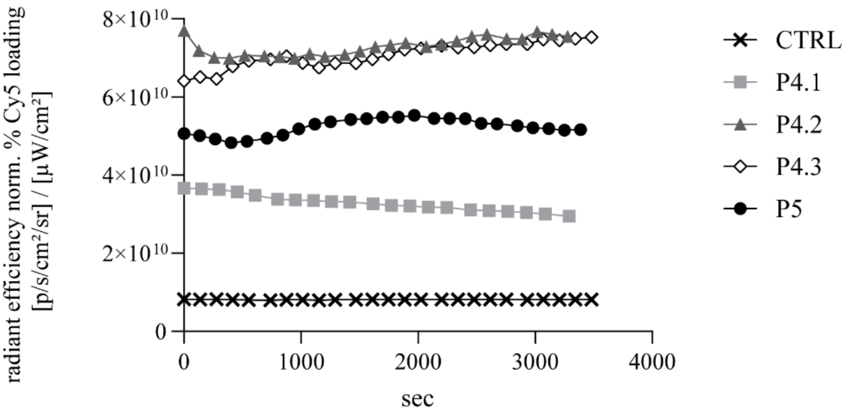

b

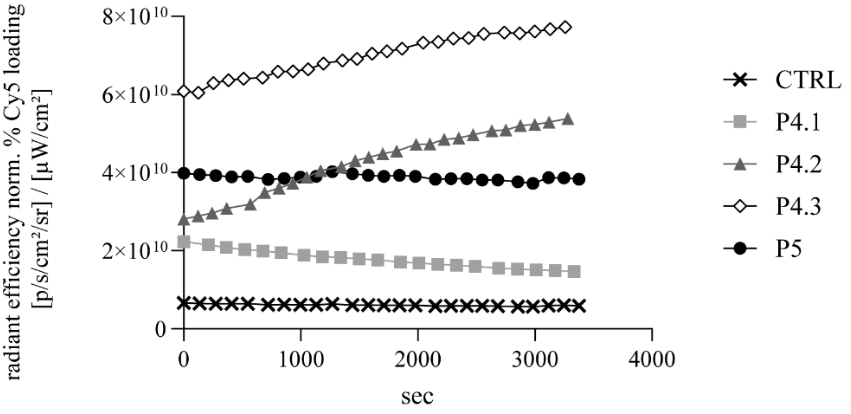

Figure S24. Kinetics of Cy5-signal within $CT26_{luc}$ tumor area after i.v. administration of Cy5-labeled bottlebrush polymers *in vivo*: Fluorescence signal in the tumor area for each bottlebrush polymer was estimated over time (60 min) by image analysis as shown in Figure S23 and normalized to the Cy5-loading of the polymers. Panels a and b show different sets of mice measured in prone position.

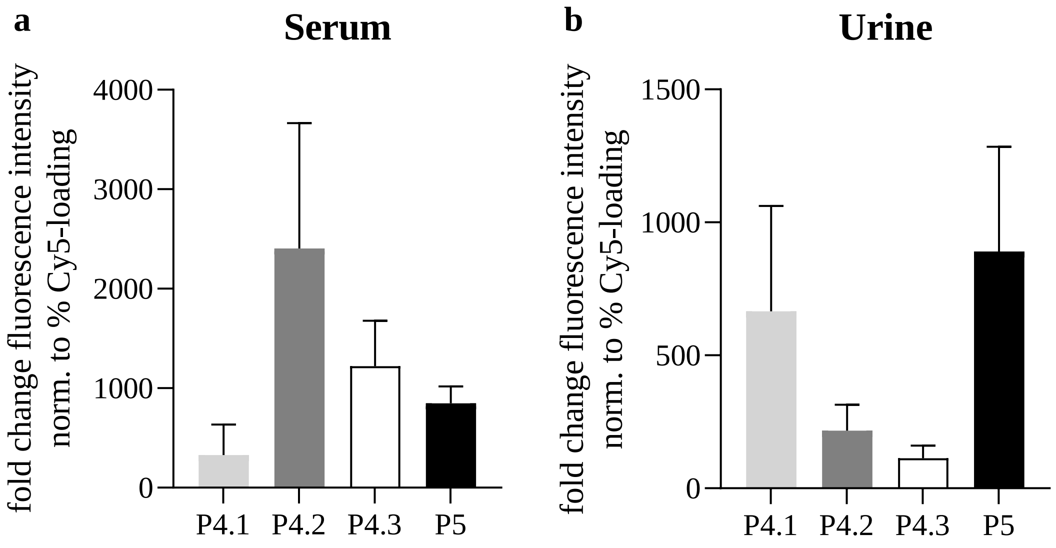

Figure S25. Pharmacokinetic studies in $CT26_{luc}$ tumor-bearing mice on a low-fluorescence diet. On day 10 mice were treated with **P4.1, P4.2, P4.**3 and **P5** (100 mg/kg i.v. dissolved in 0.9 % NaCl), followed by serum as well urine analysis for 1 h. Cy5-fluorescence intensity of serum (a) as well as urine (b) was measured by a fluorescence plate reader. Values given in the graph are the mean fluorescence intensity normalized to % Cy5 loading ± SD in quadruplicates.

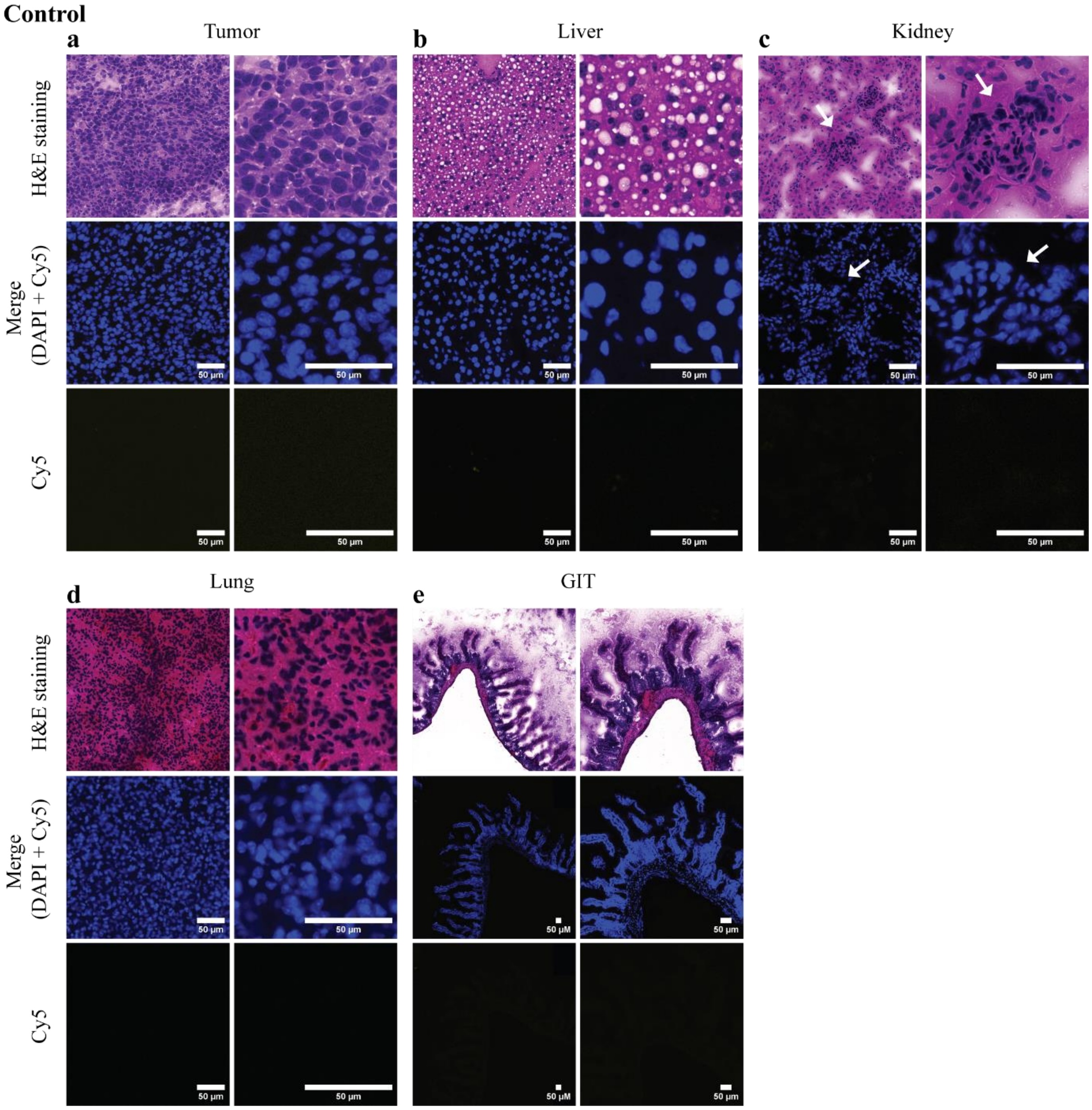

Figure S26. Representative fluorescence microscopy images of tissues collected from control mice after the IVIS measurements. Tissues were OCT-embedded and snap-frozen, followed by cutting and histological staining (H&E or DAPI). Pictures were taken by a fully automated 12 slide stage (TissueFAXS, TissueGnostics) on Zeiss observer microscope (objective magnification 20 x and 63 x; scale bar: 50 µm; yellow (polymer) and blue (nucleus)). (a) tumor, (b) liver, (c) kidney, (d) lung and (e) GIT. Arrows indicate glomeruli.

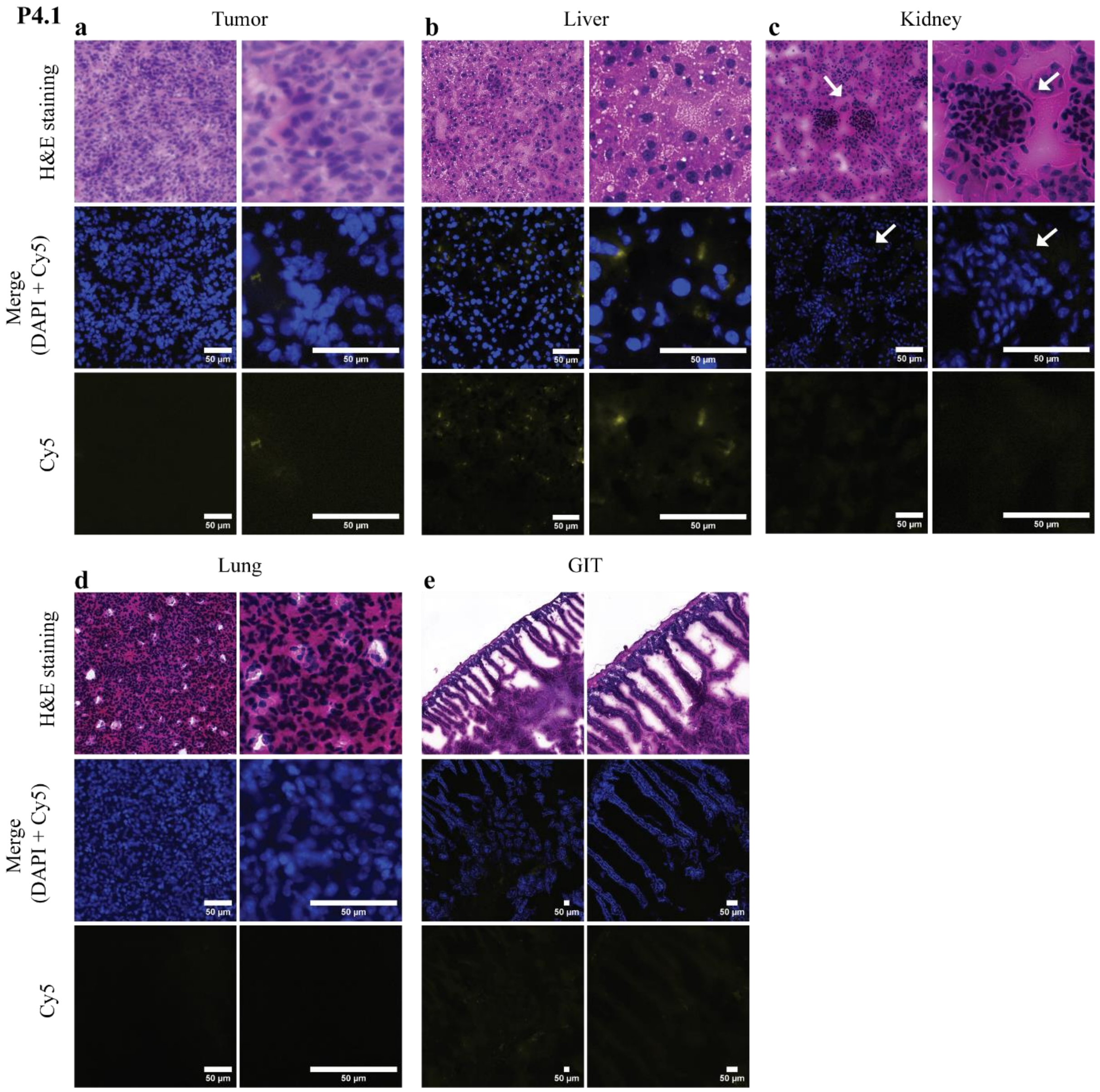

Figure S27. Representative fluorescence microscopy images of tissues collected from **P4.1**-treated mice after the IVIS measurements. Tissues were OCT-embedded and snap-frozen, followed by cutting and histological staining (H&E or DAPI). Pictures were taken by a fully automated 12 slide stage (TissueFAXS, TissueGnostics) on Zeiss observer microscope (objective magnification 20 x and 63 x; scale bar: 50 µm; yellow (polymer) and blue (nucleus)). (a) tumor, (b) liver, (c) kidney, (d) lung and (e) GIT. Arrows indicate glomeruli.

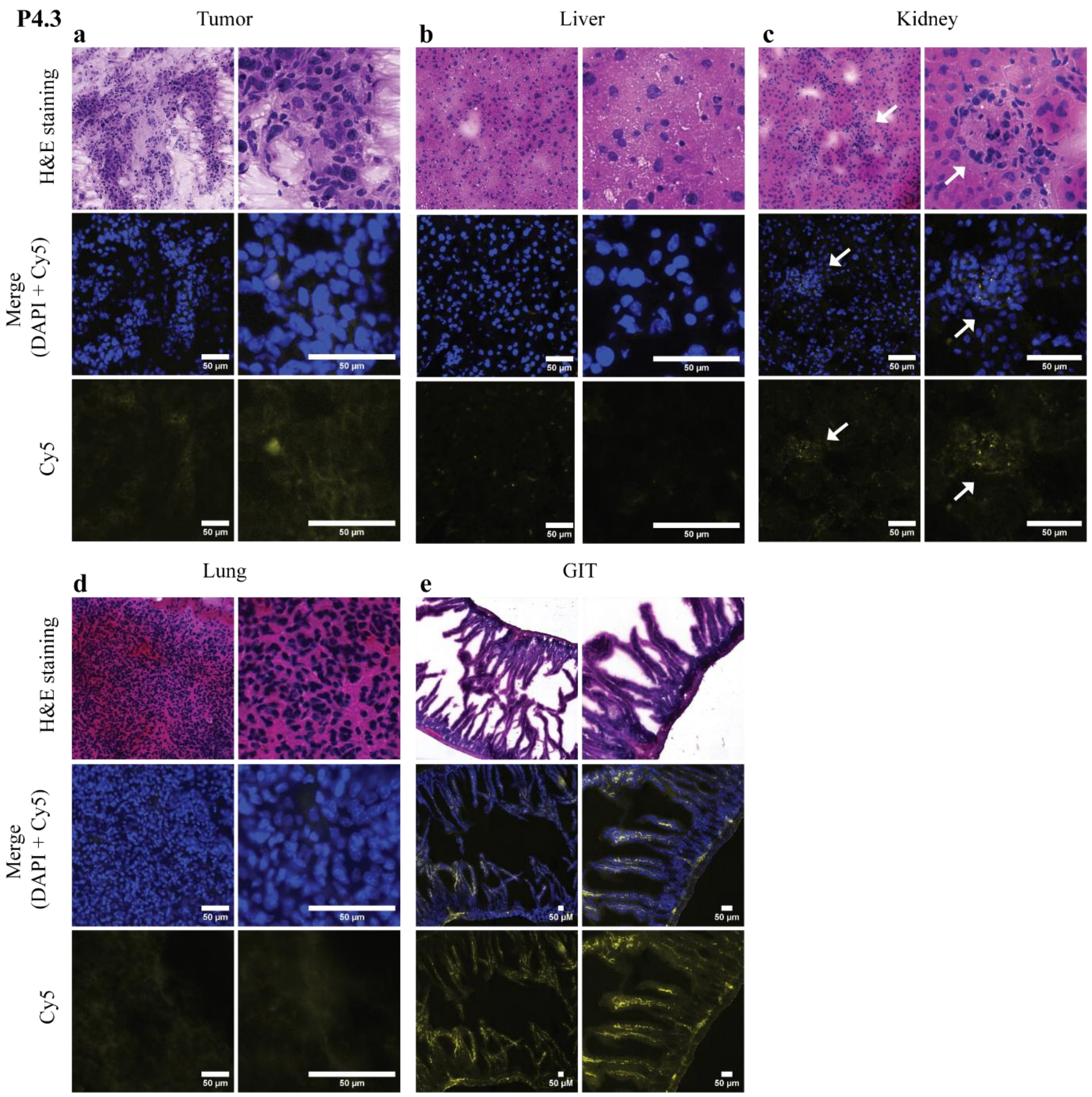

Figure S28. Representative fluorescence microscopy images of tissues collected from **P4.3**-treated mice after the IVIS measurements. Tissues were OCT-embedded and snap-frozen, followed by cutting and histological staining (H&E or DAPI). Pictures were taken by a fully automated 12 slide stage (TissueFAXS, TissueGnostics) on Zeiss observer microscope (objective magnification 20 x and 63 x; scale bar: 50 µm; yellow (polymer) and blue (nucleus)). (a) tumor, (b) liver, (c) kidney, (d) lung and (e) GIT. Arrows indicate glomeruli.

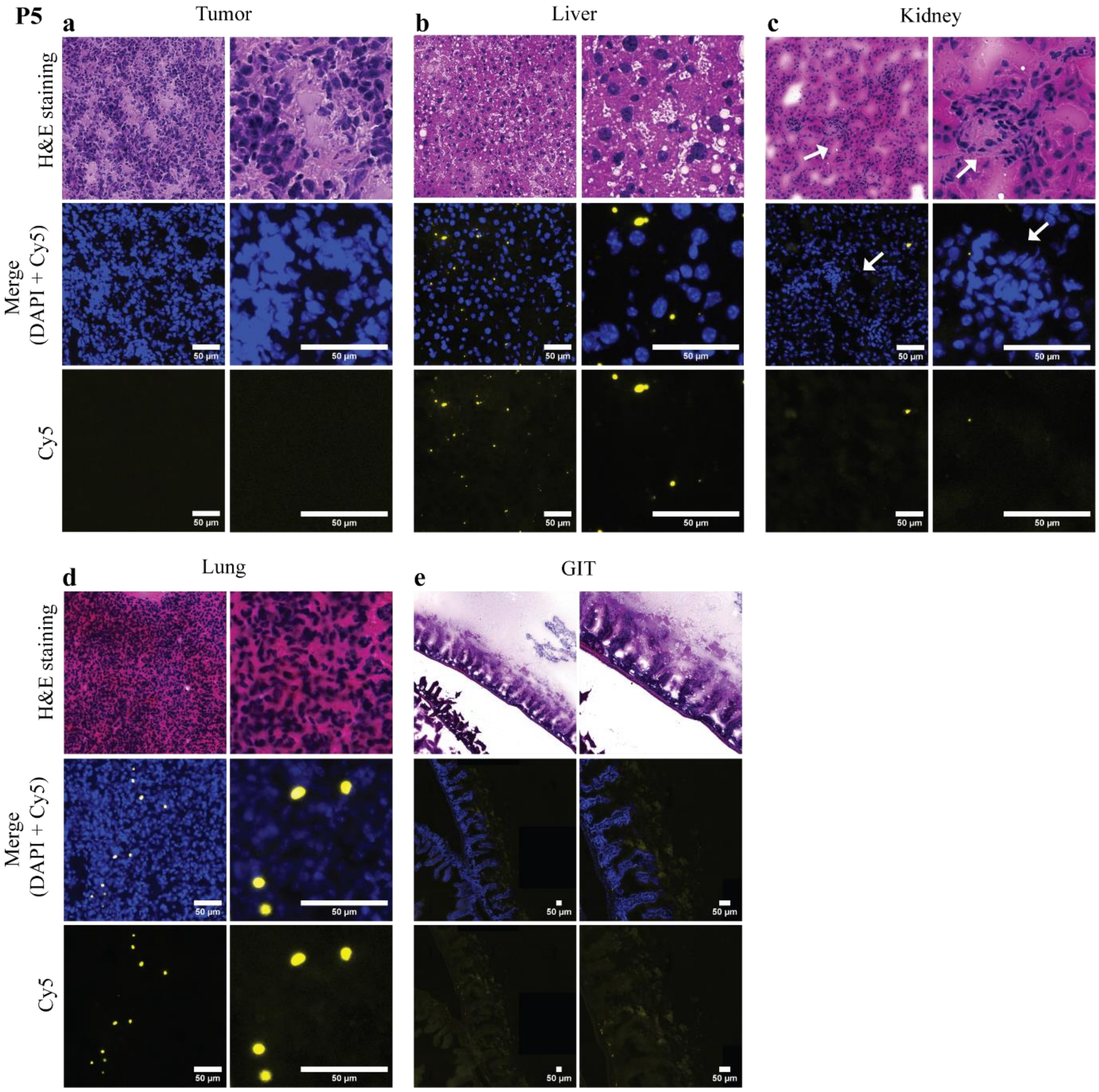

Figure S29. Representative fluorescence microscopy images of tissues collected from **P5**-treated mice after the IVIS measurements. Tissues were OCT-embedded and snap-frozen, followed by cutting and histological staining (H&E or DAPI). Pictures were taken by a fully automated 12 slide stage (TissueFAXS, TissueGnostics) on Zeiss observer microscope (objective magnification 20 x and 63 x; scale bar: 50 µm; yellow (polymer) and blue (nucleus)). (a) tumor, (b) liver, (c) kidney, (d) lung and (e) GIT. Arrows indicate glomeruli.
